# Suppressor mutations in *Mecp2*-null mice implicate the DNA damage response in Rett syndrome pathology

**DOI:** 10.1101/gr.258400.119

**Published:** 2020-04

**Authors:** Adebola Enikanolaiye, Julie Ruston, Rong Zeng, Christine Taylor, Marijke Schrock, Christie M. Buchovecky, Jay Shendure, Elif Acar, Monica J. Justice

**Affiliations:** 1 Program in Genetics and Genome Biology, The Hospital for Sick Children, Toronto, Ontario, M5G 0A4, Canada;; 2 Department of Molecular and Human Genetics, Baylor College of Medicine, Houston, Texas 77030, USA;; 3 Department of Genome Sciences, University of Washington, Seattle, Washington 98195, USA;; 4 Brotman Baty Institute for Precision Medicine, Seattle, Washington 98195, USA;; 5 Allen Discovery Center for Cell Lineage Tracing, Seattle, Washington 98195, USA;; 6 Howard Hughes Medical Institute, Seattle, Washington 98195, USA;; 7 The Centre for Phenogenomics, Toronto, Ontario, M5T 3H7, Canada;; 8 Department of Statistics, University of Manitoba, Winnipeg, Manitoba, R3T 2N2, Canada;; 9 Department of Molecular Genetics, University of Toronto, Toronto, Ontario, M5S 1A8, Canada

## Abstract

Mutations in X-linked methyl-CpG-binding protein 2 (*MECP2)* cause Rett syndrome (RTT). To identify functional pathways that could inform therapeutic entry points, we carried out a genetic screen for secondary mutations that improved phenotypes in *Mecp2*/Y mice after mutagenesis with *N*-ethyl-*N*-nitrosourea (ENU). Here, we report the isolation of 106 founder animals that show suppression of *Mecp2*-null traits from screening 3177 *Mecp2*/Y genomes. Whole-exome sequencing, genetic crosses, and association analysis identified 22 candidate genes. Additional lesions in these candidate genes or pathway components associate variant alleles with phenotypic improvement in 30 lines. A network analysis shows that 63% of the genes cluster into the functional categories of transcriptional repression, chromatin modification, or DNA repair, delineating a pathway relationship with MECP2. Many mutations lie in genes that modulate synaptic signaling or lipid homeostasis. Mutations in genes that function in the DNA damage response (DDR) also improve phenotypes in *Mecp2/Y* mice. Association analysis was successful in resolving combinatorial effects of multiple loci. One line, which carries a suppressor mutation in a gene required for cholesterol synthesis, *Sqle*, carries a second mutation in retinoblastoma binding protein 8, endonuclease (*Rbbp8*, also known as *CtIP*), which regulates a DDR choice in double-stranded break (DSB) repair. Cells from *Mecp2*/Y mice have increased DSBs, so this finding suggests that the balance between homology-directed repair and nonhomologous end joining is important for neuronal cells. In this and other lines, two suppressor mutations confer greater improvement than one alone, suggesting that combination therapies could be effective in RTT.

Mouse genetics is a powerful tool to identify molecular mechanisms that are important for disease suppression. By using a modifier screen, a dominant mutation can be isolated by its ability to alter the presentation of a recessive or dominant trait to discover genes that act in a given developmental or biochemical pathway. Modifier screens have been rare in the mouse; however, using massively parallel sequencing technologies, candidate mutations can now be efficiently identified without extensive breeding and mapping. Mutagenesis screens that focus on disease suppression may identify unrecognized pathways of pathogenesis as alternative therapeutic entry points.

Rett syndrome (RTT) is a prototype disease for which a modifier screen would be beneficial and representative for other diseases. RTT is a nearly monogenic disorder with >95% of patients carrying mutations in methyl-CpG-binding protein 2 (*MECP2*), a gene not present in invertebrates. MECP2 is central to neurological function and is associated with other diseases, including intellectual disabilities, autism, neuropsychiatric disorders, and lupus erythematosus (Neul and Zoghbi 2004; Bienvenu and Chelly 2006). The type of *MECP2* mutation does not always correlate with disease severity, in part, because of skewing of X Chromosome inactivation in heterozygous females (Shahbazian et al. 2002). Hemizygous males with truncating or loss of function mutations in *MECP2* usually die by two years of age (Bienvenu and Chelly 2006).

Mouse models recapitulate many of the pathologic features of RTT and are crucial for understanding the molecular and cellular basis (Chen et al. 2001; Guy et al. 2001). Male mice that lack *Mecp2* are normal through three weeks of age, but they develop hypoactivity, limb clasping, tremors, and abnormal breathing as early as four weeks, depending upon the allele and the genetic background (Katz et al. 2012). The symptoms become progressively worse, leading to their death at 6–12 wk. RTT has historically been considered a neurological disease (Jellinger and Seitelberger 1986). MECP2 is expressed at near histone levels in neurons, and neurons of *Mecp2*/Y mice show a number of deficits, including delayed transition into mature stages, altered expression of presynaptic proteins, and reduced dendritic spine density (Xu et al. 2014). However, MECP2's expression outside the nervous system leads to a number of systemic issues, including metabolic syndrome (Kyle et al. 2016, 2018; Ross et al. 2016).

Multiallelic contributions or environmental effects may cause the wide degree of phenotypic variation that is common to many human diseases (Enikanolaiye and Justice 2019). Rare familial cases of RTT suggest that genetic modification can alter the severity of symptoms (Ravn et al. 2011; Grillo et al. 2012), but for any rare disorder, finding the molecular basis for phenotypic modification in humans is daunting. Identifying genes that when mutated ameliorate the phenotype in a mouse model would help to focus efforts toward understanding the functions of MECP2 and how the disease ensues upon its loss. Further, identifying the genes that improve the RTT-like phenotype in mice may allow for the discovery of pathways that inform therapeutic strategies in humans. In a preliminary dominant suppressor mutagenesis screen using the supermutagen *N*-ethyl-*N*-nitrosourea (ENU), we isolated five modifier loci, named suppressor of methyl CpG binding protein (*Sum) 1–5*, which suppress or ameliorate the phenotype in *Mecp2*/Y male mice (Buchovecky et al. 2013). We previously reported that the lesion responsible for disease amelioration at the *Sum3*^*Jus*^ locus is a stop codon mutation in a rate-limiting enzyme in cholesterol biosynthesis, squalene epoxidase (*Sqle*). This finding led to the discovery that cholesterol and lipid homeostasis is perturbed in *Mecp2* male and female mice, and that statin drugs ameliorate symptoms as well (Buchovecky et al. 2013). This discovery suggested that finding additional modifiers would lead to a better understanding of *Mecp2*’s functions and how its mutation could lead to pathological changes. Our overarching goal was to identify a large number of suppressors such that a list of potential therapeutic targets could be developed for RTT.

## Results

### Two suppressor screens produce 106 founders with trait improvement

**Figure 1.**
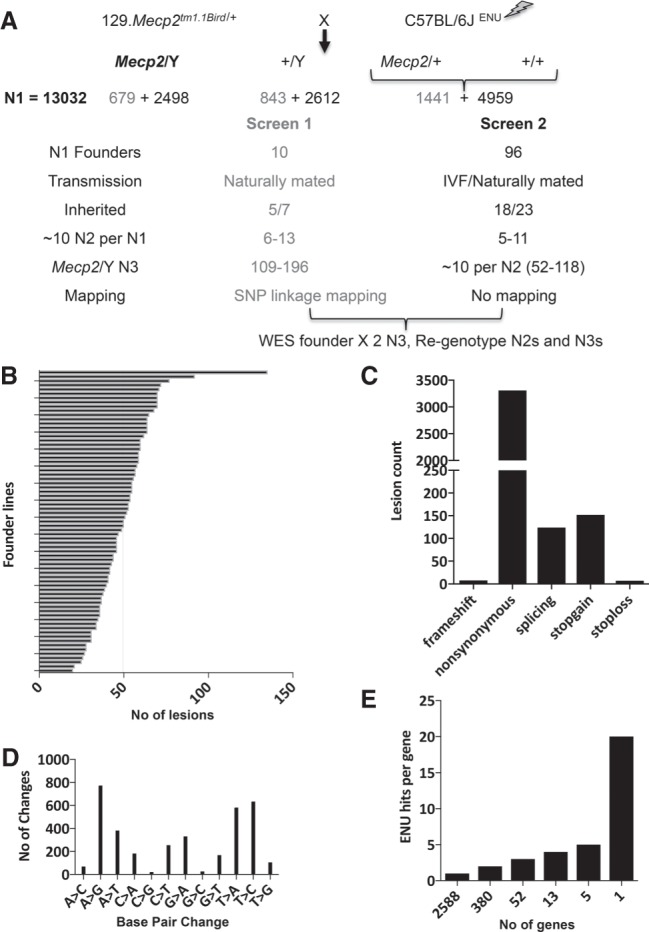
Overview of two dominant suppressor ENU mutagenesis screens in a *Mecp2^tm1.1Bird^* mouse model. (*A*) Male C57BL/6J male mice were treated with ENU and mated to 129.*Mecp2^tm1.1Bird^*^/+^ females. Numbers obtained from screen 1, already published, are in gray, and screen 2 in black. A subset of N1 male founder mice (N1) carrying the mutant *Mecp2* allele and showing suppression of disease phenotypes were bred (naturally or by IVF) for inheritance. N2 offspring were mated to 129.*Mecp2^tm1.1Bird^*^/+^ females or 129S6/SvEvTac males to generate N3s, of which DNAs from at least two animals from different N2 families that showed trait improvement were whole-exome sequenced (WES). N3 offspring were genotyped to analyze for linkage or association. (*B*) The WES was analyzed for 71 founder lines, producing 3601 potentially causative variants. Variants ranged in number from 20 to 135, with a mean of 50. (*C*) The types of ENU-induced lesions generated were mostly nonsynonymous missense mutations (3310, 92%), followed by nonsense mutations (152, 4%) and splicing mutations (124, 3%). Frameshift mutations or the loss of a STOP codon accounted for the remainder (1%). (*D*) The most common types of mutations induced by ENU were A to G and T to C transition mutations (22% and 18%, respectively), closely followed by transversions (A to T [11%] and T to A [16%]), whereas the fewest were C to G and G to C transversions (<1%). (*E*) The majority of genes with new ENU-induced lesions were unique, with 2588 genes carrying only one mutation. A single gene, titin (*Ttn*), had 20 alleles.

ENU-treated C57BL/6J males were bred to female 129.*Mecp2*^tm1.1Bird/+^ mice (Fig. 1A). Male offspring in the first generation (N1), asymptomatic at weaning, were genotyped for presence of mutant *Mecp2* and examined for amelioration of disease phenotypes by a dominant mutation. In total, 3177 relevant gametes were screened for phenotype improvement. The preliminary screen 1 produced 2963 animals of which 679 were *Mecp2*/Y (Fig. 1A, gray; Buchovecky et al. 2013). This number is close to one genome's equivalent, because the mutation rate for the 3 × 100 mg/kg dose of ENU is about one new mutation per gene in every 655 gametes screened (Hitotsumachi et al. 1985). In screen 1, 10 lines showing suppression of traits were identified, but only six of these bred naturally and one (1527) was reconstituted by in vitro fertilization (IVF) of fresh sperm. A scoring system was used to individually assess limb clasping, tremors, body weight, and activity. In general, improvement of these scores indicated improved longevity as evidenced by the long lifetime of the founders and their offspring (Buchovecky et al. 2013).

In screen 2, an additional 10,069 animals, of which 2498 were *Mecp2*/Y, produced 96 additional N1 founder males that showed suppression of one or more traits. In screen 2, sperm from each founder was frozen, and of 23 lines mated, nine bred naturally before they were sacrificed, whereas 14 were reconstituted using IVF of frozen sperm. In screen 2, all N1 mice were evaluated weekly starting at P35 for four subjective health parameters that have previously been noted in *Mecp2/Y* mice (Guy et al. 2001): hindlimb clasping, tremors, activity, and general body condition. Muscle tone was also assessed, because it was also an important indicator of health. Each subjective parameter was scored out of 2, whereby 0 indicated the health trait was equivalent to wild type, 1 if intermediate, or 2 if severe. Body weights were also obtained weekly and were scored 0 if <29 g, 1 if between 30 and 34 g, and 2 if >35 g. These five subjective health parameters and quantitative parameter body weight were totaled to obtain a score out of 12. In screen 2, animals were classified as improved if their summed health score totaled <8 at 8 wk of age, and they were maintained generally until 12–14 wk, when they were sacrificed for sperm cryopreservation. N3 animals were evaluated using the same criteria. The *Mecp2*/Y animals from the strains maintained at the two institutions had different life spans, possibly owing to differing pathogen status. Therefore, *Mecp2*/Y animals from screen 1 were classified improved if they lived ≥14 wk, while those from screen 2 were classified improved if they lived ≥10 wk and had improved scores on assessed health traits.

### Whole-exome sequencing identifies candidate genes in founder males

When the first modifiers were isolated in screen 1, WES was not yet available, so the first line was sequenced by custom capture resequencing of a mapped region (line 352). WES was carried out on lines 856, 895, 1395, and 1527, but the sequence was analyzed only in the regions with positive LOD scores obtained by mapping, and only one candidate gene, *Sqle*, from line 895 was reported (Buchovecky et al. 2013). The WES of 69 of the founder N1 males from the second screen was analyzed for all potential contributing mutations throughout the genome (Supplemental Table S1). Naturally occurring single-nucleotide polymorphisms (SNPs) that were specific to the 129S6/SvEv and C57BL/6J strains were filtered by comparing with parental and all sequenced founder male genomes, and variants predicted to be tolerated were excluded. The remaining potentially causative 3601 variants ranged in number from 20 to 135 in each N1 founder male, with a mean of 50 (Fig. 1B). These variants were largely nonsynonymous missense mutations (92%) (Fig. 1C), which were primarily transition mutations (A to G and T to C) (Fig. 1D). Each of 2588 genes had only one allele found in the N1 males, each of 380 genes had different alleles in two lines, 52 genes had three alleles, 13 genes had four, five genes had five, and a single large gene, titin (*Ttn*), had 20 alleles (Fig. 1E).

### Confirming the candidate lesions shows complex inheritance of traits

The inheritance of traits was determined by mating 30 N1 founders from both screens (seven from screen 1 and 23 from screen 2) to 129.*Mecp2*^/+^ females. Their N2 female offspring, carrying the *Mecp2* mutation inherited from their father, were mated again to 129S6/SvEvTac males, whereas N2 male offspring were mated to 129.*Mecp2*^/+^ females. In screen 1, each line was mapped to narrow the suppressor locus to a chromosomal location using SNP panels before sequencing (Neuhaus and Beier 1998; Moran et al. 2006). In screen 2, SNPs were not used for mapping. When possible, up to 10 N2 offspring from each family were mated to produce approximately 10 *Mecp2*/Y N3 offspring, which were assessed for phenotype and aged. If the modifier was inherited in a Mendelian fashion, 50% of the N2 animals should have inherited the trait and 50% should pass it on to their N3 offspring; thus, only 25% of the total N3 offspring are expected to inherit the suppressor trait (Table 1). Of 30 lines, 23 showed evidence for inheritance of disease improvement (Supplemental Table S2). Nineteen of the 23 lines segregated modifier loci in a non-Mendelian fashion, suggesting involvement of more than one locus (Table 1). In lines 856 and 895, the pattern of inheritance suggested that either locus conferred improvement, whereas the inheritance of both loci suggested additive health improvement. In contrast, although all families in line 520 had at least one animal with trait improvement, none showed greatly improved health scores, suggesting that the two loci may have influenced health traits independently. Seven lines, 137, 591, 722, 933_15N, A_87N, A_120N, and A_134L, were not solved because so few *Mecp2*/Y N3 animals showed trait improvement (fewer than eight), possibly because two or more loci must be inherited together to see an effect. It is also possible that a genetic combination resulted in decreased health or longevity, rather than improvement.

**Table 1.**
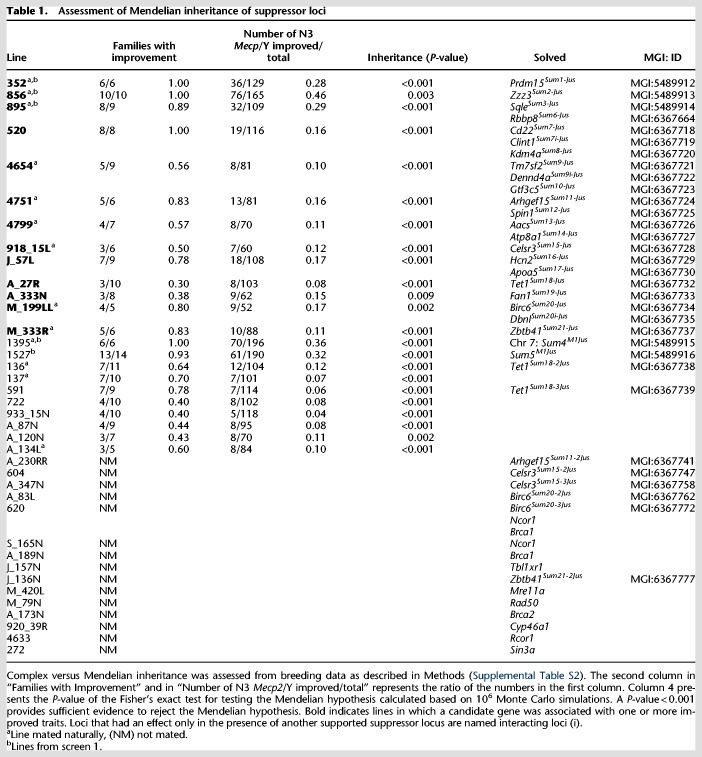
Assessment of Mendelian inheritance of suppressor loci

To identify candidate genes in screen 2, the exome from a minimum of two N3 offspring that showed the largest degree of improvement and were from different N2 parents was also sequenced. Lesions that occurred in the N1 founder and these two offspring were regenotyped in individuals from families that inherited trait improvement (Fig. 1A; Supplemental Table S2). Common SNPs identified in WES were used to genotype N3 animals to confirm the location after candidate loci were identified.

Statistical analysis of candidate genes shows support for a solution in 13/23 lines (Tables 1, 2; Supplemental Table S3). Within these 13 lines, 22 genes are supported by association analysis as candidates for suppression of a variety of traits. Eight additional lines carry alleles of these 22 candidate genes, and 11 lines carry mutations in a related pathway member or *Mecp2* corepressor complex member. Some lines carry multiple associative mutations, thus, candidate genes have been identified in 30 of the lines (Tables 1, 2). The screen 2 loci that are supported by mating and association analysis are named *Sum 6–21* (Table 1). Candidate genes are identified for two of the lines from screen 1 (*Prdm15^Sum1-Jus^* and *Zzz3^Sum2-Jus^*), and a second suppressor was found in line 895 from screen 1 (*Rbbp8^Sum6-Jus^*).

### Candidate genes fall into similar functional pathways

**Figure 2.**
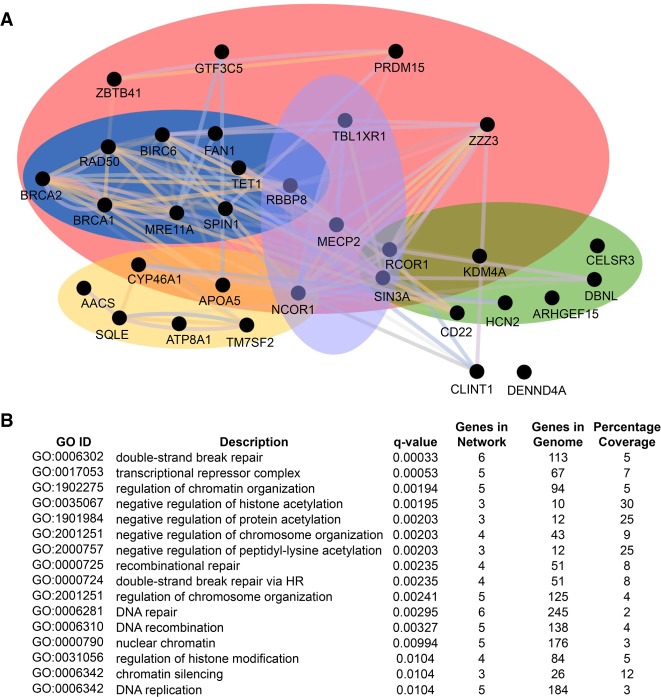
A functional network analysis of proteins encoded by candidate genes. (*A*) A network enrichment map showing the relationships between proteins with supported or pathway lesions. Proteins are represented as black dots or nodes, and the relationships between them are represented as colored lines or edges, in which purple lines denote coexpression, pink lines denote physical interactions, orange lines denote predicted interactions, blue lines denote colocalization, and green lines denote shared protein domains. Further clustering (indicated by colored ovals) revealed that most of the proteins group into the broad categories of lipid metabolism and homeostasis (yellow), synaptic function (green), and DNA damage response (blue). Several proteins are also involved in transcriptional repression (purple). Sixty-three percent are included in the broad category regulation of DNA activity (pink). (*B*) The most enriched Gene Ontology (GO) terms in this network, as determined by their false discovery rate (FDR)-adjusted *q*-value, are double-strand break repair, transcriptional repressor complex, negative regulation of histone acetylation, and regulation of chromatin organization. The percentage coverage indicates the proportion of genes within a given GO ID that are also present in the network.

Genes with supported or pathway lesions were integrated into biological networks using Cytoscape (v3.7) (Shannon et al. 2003) and its accompanying applications GeneMANIA (v3.5.1) (Warde-Farley et al. 2010) and clusterMaker (1.3.1) (Morris et al. 2011). A functional enrichment map (network) generated by coexpression, shared protein domains, physical interactions or predictions was further clustered into biological pathways, confirming that most of the genes fall into three major pathways; lipid homeostasis, synaptic function, and DNA damage (Fig. 2). Gene Ontology (GO) analysis revealed that the top 16 enriched terms in this network included MECP2-associated biological processes involved in transcriptional corepressor activity and chromatin-associated functions. However, it also revealed novel GO terms such as DNA recombination, double-strand break repair, and regulation of DNA metabolic processes. Thus 20/32 or 63% of the genes cluster into the general category of “Regulation of DNA Activity,” which is expected for a functional relationship with MECP2. The genes with unknown function (*Clint1, Dennd4a*) fall outside the functional pathways.

#### Chromatin structure and transcriptional regulation

Several candidate genes are predicted to alter chromatin structure: These include PR domain containing 15 (*Prdm15*), zinc finger ZZ domain containing 3 (*Zzz3*), and lysine (K)-specific demethylase 4A (*Kdm4a*). The PR domain proteins, many of which have histone methyltransferase activity and others that recruit methyltransferases to DNA, play roles as molecular switches in many developmental processes (Fog et al. 2012; Hohenauer and Moore 2012; Mzoughi et al. 2017). ZZZ3 is a part of the Ada-Two-A-containing (ATAC) histone acetyltransferase complex, which widely regulates gene expression (Mi et al. 2018). KDM4A is important for the structure of heterochromatin during embryonic development, and it influences neuropathic pain through brain derived neurotrophic factor (BDNF) expression (Sankar et al. 2017; Zhou et al. 2017). The corepressors *Ncor1,* transducin (beta)-like 1X-linked receptor 1(*Tbl1xr1*), *Rcor1*, and *Sin3a* are transcriptional repressors that act with MECP2, yet missense mutations in these genes occurred in lines that were not mated, so they are included in Table 2 but are not given *Sum* designations.

**Table 2.**
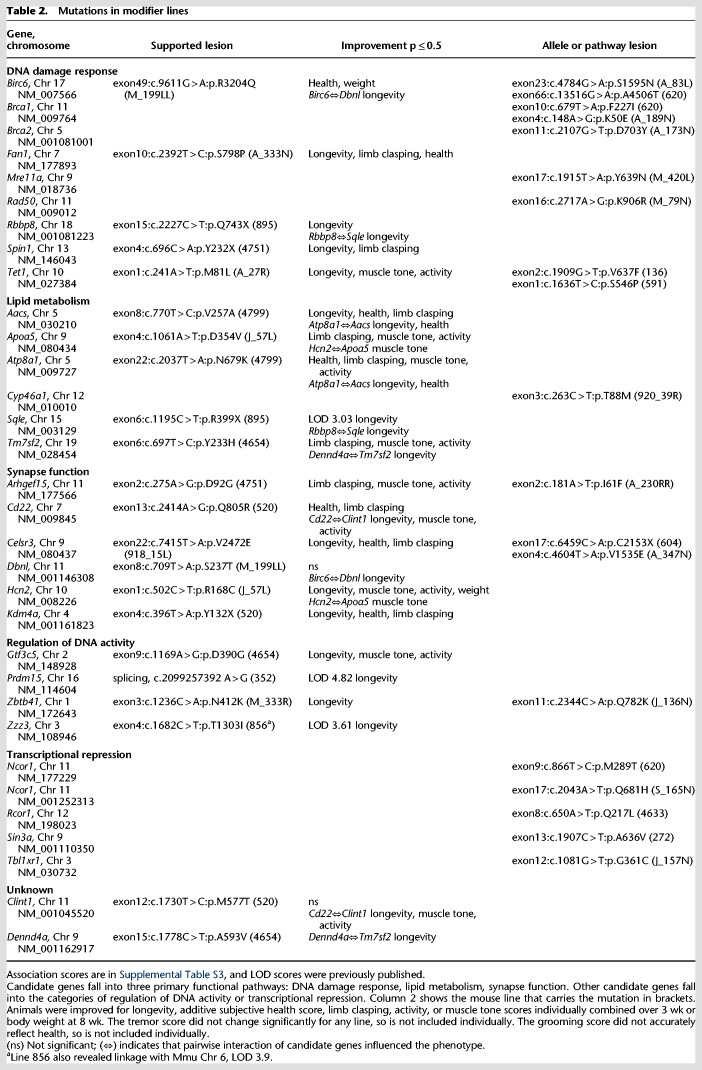
Mutations in modifier lines

#### Lipid homeostasis

Four mutations (*Aacs, Apoa5, Atp8a1, Tm7sf2*) support previous publications that lipid metabolism is a primary pathway for pathogenesis (Buchovecky et al. 2013; Justice et al. 2013; Segatto et al. 2014; Kyle et al. 2016). The founding lesion in this pathway occurred in *Sqle*, a rate-limiting enzyme in cholesterol synthesis. MECP2 anchors a protein complex containing the master metabolic regulator nuclear receptor co-repressor 1 (NCOR1) to DNA. When MECP2 is absent, NCOR1 does not suppress its lipid synthesis targets, including *Sqle,* leading to lipid accumulation in the brain and liver of *Mecp2*-mutant males and females, resulting in metabolic syndrome (Buchovecky et al. 2013; Kyle et al. 2016). Acetoacetyl-CoA synthetase (*Aacs*) is another target of the NCOR1 corepressor complex (Knutson et al. 2008), which allows ketone bodies to be used as an energy source (Hasegawa et al. 2012a,b). Transmembrane 7 superfamily member 2 (*Tm7sf2*) functions just downstream from *Sqle* in the cholesterol biosynthesis pathway, and its function is linked to liver X receptor (LXR) and NF-kB signaling, suggesting roles in both lipid homeostasis and inflammation (Bellezza et al. 2013). Apolipoprotein A-V (*Apoa5*) is a key regulator of triglyceride metabolism (Gonzales et al. 2013). ATPase, aminophospholipid transporter (APLT), class I, type 8A, member (*Atp8a1*) is part of an ATPase-coupled membrane complex involved in vesicle trafficking and the transport of aminophospholipids (Paterson et al. 2006). A mutation in line 920_39R occurred in the neuron-specific cytochrome P450, family 46, subfamily a, polypeptide 1 (*Cyp46a1*), which produces 24*S*-OHC, a molecule essential for cholesterol turnover and a biomarker for abnormal cholesterol homeostasis in the *Mecp2* brain (Lund et al. 2003; Buchovecky et al. 2013).

#### Synapse function

Mutations that affect synaptic function are expected from previous studies of RTT patients and *Mecp2-*mutant mice (Chao et al. 2007; Shepherd and Katz 2011; Bellini et al. 2014). A mutation in cadherin, EGF LAG seven-pass G-type receptor 3 (*Celsr3*) was supported by mating and association analysis, and two additional lines (604, A_347N) carried alleles. CELSR3 has a role in glutamatergic synapse formation and neuronal development (Feng et al. 2016; Thakar et al. 2017; Wang et al. 2017). Moreover, *Celsr3* is a target of the REST/Co-REST complex, which recruits other proteins including histone deacetylases (HDACs) and the transcriptional regulator SIN3A to mediate long-term gene silencing of target genes in neurons (Ballas et al. 2005; Mandel et al. 2011; Monaghan et al. 2017; Hwang and Zukin 2018). Similarly, drebrin-like (*Dbnl*) is a REST target involved in vesicular trafficking and synapse formation in neurons (Haeckel et al. 2008). Although line 4633 was not mated, it carries a missense mutation in co-REST, *Rcor1,* which binds and corepresses target genes with MECP2 (Ballas et al. 2005). Other candidate genes that are predicted to function at the synapse are the hyperpolarization-activated, cyclic nucleotide-gated K+ 2 (*Hcn2*) and the Rho guanine nucleotide exchange factor (GEF) 15 (*Arhgef15*). *Hcn2* is a target of cAMP that regulates neuronal extension (Mobley et al. 2010), and mice that lack *Arhgef15* have increased excitatory synapse formation (Margolis et al. 2010). CD22 antigen (*Cd22*) is a canonical B-cell receptor that clusters in synaptic function because mutations in *Cd22* increase the ability of microglia to clear myelin debris and remodel dendrites (Pluvinage et al. 2019).

#### DNA damage response

Another group of mutations affects the DNA damage response (DDR). Three lines carried mutations in tet methylcytosine dioxygenase 1 (*Tet1*). TET1 modifies methylated CpG dinucleotides to 5-OH methylated bases in the first step of demethylation by base excision repair (BER) (Weber et al. 2016). *Tet1* plays a role in chromosomal stability and telomere length in embryonic stem cells through methylation (Yang et al. 2016). Although only one of the *Tet1* lesions is supported by mating and association analysis in line A_27R, all three are missense mutations that lie in evolutionarily conserved amino acids near the CpG binding domain (Supplemental Fig. S1A). Mutations in the E2/E3 ubiquitin ligase baculoviral IAP repeat-containing 6 (*Birc6*, also called BRUCE) were also found in three lines (Supplemental Fig. S1B), and one in line M_199L was confirmed by mating and association analysis. BIRC6 stabilizes TRP53 in a first step toward inhibiting apoptosis (Ren et al. 2005), but BIRC6 also regulates the BRIT1-SWI-SNF double-strand break (DSB) response (Ge et al. 2015). BIRC6-depleted cells display reduced homologous recombination repair, and *Birc6*-mutant mice show repair defects and genomic instability (Lotz et al. 2004; Ren et al. 2005). The mutation in Spindlin 1 (*Spin1*) lies within its third TUDOR-like domain (Supplemental Fig. S2), which binds methylated histone H3, a mark associated with DDR (Chew et al. 2013; Choi et al. 2017; Shanle et al. 2017).

**Figure 3.**
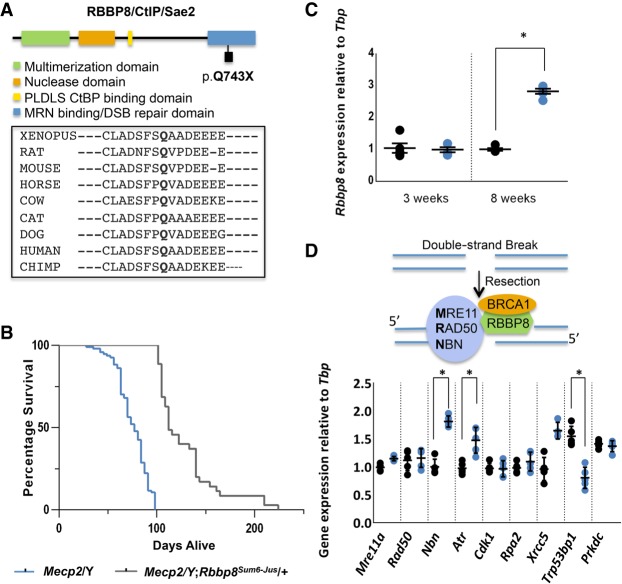
The DNA damage response (DDR) pathway is implicated in a *Mecp2*/Y mouse model of Rett syndrome. (*A*) An ENU-induced nonsense mutation occurs at position p.Q743X within the critical C-terminal double-strand break (DSB) repair domain (blue) of RBBP8, also called CtBP interacting protein CtIP or SAE2 in yeast. Amino acid alignments generated using Clustal Omega show that this glutamine residue is well conserved among organisms from *Xenopus* to human. (*B*) *Mecp2*/Y;*Rbbp8^Sum6-Jus^*^/+^ mice (gray) have increased longevity (*n* = 35, Median survival 112 d) when compared to *Mecp2*/Y mice (blue) (*n* = 94, Median survival 77 d) without secondary mutations (*P* < 0.0001 by Mantel-Cox test). (*C*) *Rbbp8* transcripts are elevated in symptomatic at 8 wk *Mecp2*/Y (blue) brain compared to +/Y (black), but unchanged at 3 wk. Results are representative of three independent experiments (*P* < 0.01 by the two-sample Student's *t*-test, +/Y: *n* = 5, *Mecp2*/Y: *n* = 5); error bars represent SEM. (*D*) Double-strand break repair by homologous recombination involves the recruitment of RBBP8 to the site of the break in an MRN/BRCA1-dependent manner. RBBP8 partners with BRCA1 to initiate DNA resection. Transcript levels of genes involved in HR, nibrin (*Nbn*) and ataxia telangiectasia and Rad3 related (*Atr*) are elevated at 8 wk in *Mecp2*/Y (blue) brain, whereas NHEJ gene transformation related protein 53 binding protein 1 (*Trp53bp1*) is decreased compared to +/Y (black) (*P* < 0.05 by the two-sample Student's *t*-test, +/Y: *n* = 5, *Mecp2*/Y: *n* = 5); error bars represent SEM.

Additional mutations lie in components of the DSB repair pathway. A mutation in retinoblastoma binding protein 8, endonuclease (*Rbbp8*) occurred in exon 15, which results in a premature stop codon that predicts a truncated protein in the critical C-terminal domain (Fig. 3A). RBBP8 acts with BRCA1 to regulate the DSB repair choice between homologous recombination directed repair (HDR) or nonhomologous end joining (NHEJ) (Huertas 2010; Escribano-Díaz et al. 2013), supporting the initial steps of DNA resection and recruiting other DNA repair proteins in dividing cells (Makharashvili et al. 2014). When RBBP8 is absent, NHEJ is the preferred choice for DDR repair. A survival curve shows that *Mecp2*/Y mice carrying the *Rbbp8* suppressor mutation show significantly improved longevity (Median survival of 112 d for *Mecp2*/Y*;Rbbp8*^+/−^ vs. 77 d for *Mecp2*/Y*;Rbbp8*^+/+^) (Fig. 3B). Moreover, *Rbbp8* expression is increased in *Mecp2*/Y mouse brain at a symptomatic time point of 8 wk (Fig. 3C). Together, these data suggest that elevated RBBP8 in *Mecp2*-null mice may cause pathology that is ameliorated by reducing the amount of the protein.

Previous reports show that mesenchymal and neural stem cells derived from *Mecp2*/Y mice have elevated DSBs (Squillaro et al. 2010; Alessio et al. 2018). DSBs are initially detected by the MRE11-RAD50-NBN (MRN) sensor complex, which activates the ATM or ATR kinases, leading to the phosphorylation of several downstream targets (Falck et al. 2012). RBBP8 also plays a role in the removal of topoisomerase II (TOP2)-induced DNA DSBs, where it partners with BRCA1 to initiate DNA resection before repair (Fig. 3D; Aparicio et al. 2016). Many other factors are involved in the resolution of the DSB, including BRCA2 and FANCD2/FANCI-associated nuclease 1 (FAN1) (Orthwein et al. 2015). Two mutations in *Brca1* and one in each of *Mre11a*, *Rad50*, *Fan1*, and *Brca2* were also observed (Table 2; Supplemental Fig. S2). To examine the extent of involvement of the DDR pathway, the expression of several components of the HDR (*Mre11a, Rad50, Atr, Nbn, Cdk1, Rpa2*) and NHEJ (*Xrcc5, Trp53bp1, Prkdc*) pathways were also assessed in the brains of 8-wk-old *Mecp2*/Y and +/Y mice. Expression of *Nbn*, as well as *Atr*, which transduces signals from the MRN complex, increased in whole *Mecp2*/Y mouse brain. Conversely, the expression of *Trp53bp1* decreased (Fig. 3D). TRP53BP1 functions in a manner antagonistic to RBBP8, blocking resection to promote NHEJ (Escribano-Díaz et al. 2013).

### Combinatorial mutations that improve health fall into different pathways

**Figure 4.**
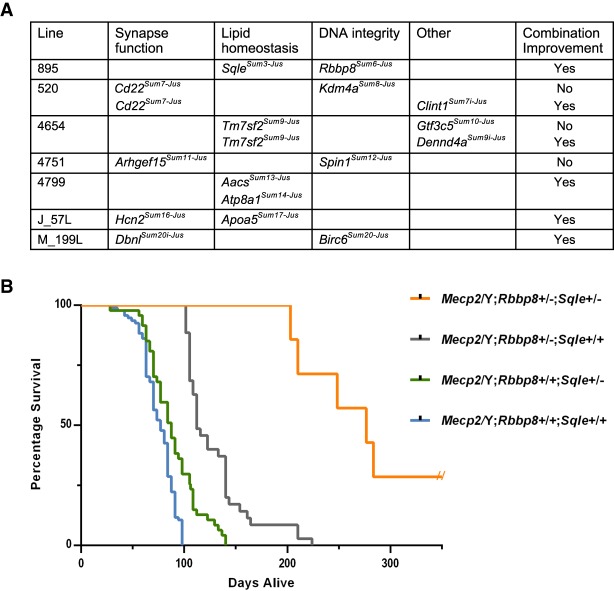
Combinatorial effects of multiple mutations on health and longevity. (*A*) Multiple lines (895, 520, 4654, 4751, 4799, J_57L, and M_199L) carry lesions in more than one gene that either independently or additively improve health. *Kdm4a* and *Cd22* in line 520, *Tm7sf2* and *Gtf3c5* in line 4654, and *Arhgef15* and *Spin1* in line 4751 show independent effects on health, whereas the other mutations show positive combinatorial effects on *Mecp2*/Y health. (*B*) *Mecp2*/Y mice from line 895, carrying mutations in *Sqle* and/or *Rbbp8*, show increased longevity when both mutations are present (*Mecp2*/Y;*Rbbp8*^+/−^;*Sqle*^+/−^; *n* = 7, median survival 277 d) when compared to *Mecp2*/Y mice carrying either mutation alone (*Mecp2*/Y;*Rbbp8*^+/−^;*Sqle*^+/+^; *n* = 35, median survival 112 d or *Mecp2*/Y;*Rbbp8*^+/+^;*Sqle*^+/−^; *n* = 47, median survival 88 d). For *Mecp2*/Y;*Rbbp8*^+/+^;*Sqle*^+/+^; *n* = 94, the median survival was 77 d. (*P* < 0.0001 by Mantel-Cox test, for each comparison of *Mecp2*/Y;*Rbbp8*^+/+^;*Sqle*^+/+^ to any of the three groups). These data include the founder 895, N3 animals (genotyped for both *Rbbp8* and *Sqle*) and all of the N5 animals generated.

Association analysis of pairwise gene–gene interactions reveals six lines in which the presence of more than one mutation confers increased improvement in individual health traits (Fig. 4A; Supplemental Tables S2, S3). In line 520, the improvement segregated independently, suggesting two suppressors, so four N3 animals instead of two were sequenced. Consistently, mutations in both *Cd22* and *Kdm4a* independently improved traits, but did not further improve traits when inherited together. However, the combined *Cd22* and *Clint1* mutations improved activity and muscle tone, although *Clint1* did not improve traits when inherited alone. Similarly, a mutation in *Gtf3c5* in line 4654 improved longevity, activity, and muscle tone independently of *Tm7sf2*. The mutation in *Tm7sf2* improved limb clasping, activity, and muscle tone, yet longevity was not improved unless the animals also carried a mutation in *Dennda*. *Dbnl* did not improve phenotypes alone in line M_199L, yet improved longevity when a mutation in *Birc6* was present. The combination of mutations in *Hcn2* and *Apoa5* in line J_57L is associated with improved muscle tone. In most cases, the associated genes belonged to different pathways (Fig. 4A). The exception is line 4799 where the combination of *Aacs* and *Atp8a1* improves longevity and health, but both genes are predicted to function in different aspects of lipid homeostasis–namely, energy utilization and lipid trafficking.

The founder of line 895 showed extreme longevity and was sacrificed at the age of 14 mo. The pattern of inheritance of line 895 indicated that he carried two different suppressor mutations (Table 1). One, a nonsense mutation in *Sqle*, was first identified by linkage (Chromosome 15, LOD 3.03). N3 males from line 895 that had a longer life were bred to segregate and identify the second locus by backcrossing again to 129S6/SvEv animals to generate N4 and N5 generations (Supplemental Table S2). Genotyping and segregation analysis revealed that a nonsense mutation in *Rbbp8* (Chromosome 18) was consistent with trait improvement. Subsequent genotyping of animals from line 895 for *Sqle* and *Rbbp8* showed that although either mutation alone was associated with improved longevity, the presence of both mutations conferred extreme longevity (Fig. 4B). Seven animals from the N3 and N5 generations inherited both loci, and all lived longer than 29 wk, with a range of 29–116 wk (203–812 d). These data suggest that combining mutations from different pathways may further improve health in *Mecp2/Y* mice.

## Discussion

Genetic modifier screens are mainly carried out in fruit flies, worms, yeast, and bacteria to discover genes that are members of a developmental or biochemical pathway. The suppressor screen reported here represents the largest screen yet carried out in the mouse, and, by using massively parallel sequencing and association analysis, the screen identifies the largest number of candidate genes. Applications of modifier screens in the mouse are powerful (Carpinelli et al. 2004; Matera et al. 2008; Westrick et al. 2017). Even so, the mutations are identified based on their ability to suppress or enhance a mutant phenotype. Consequently, at least two mutations must be segregated to follow the phenotype, requiring an extensive amount of breeding. In the screen reported here, most lines showed evidence for complex trait inheritance, predicting that more than one modifier segregated. In a recent published modifier screen for thrombosis, very few candidate genes were identified, likely because of the lack of power in finding linkages using standard quantitative trait mapping strategies (Tomberg et al. 2018). Here, statistical tests that assessed association instead of linkage were used to identify multiple interacting loci in six lines, three of which had an effect only in the presence of other modifiers. These data show the power of ENU for inducing and identifying complex genetic interactions. However, the screen has limitations, of which the most relevant to RTT is the restriction of assessments to male mice, rather than females, whose variable penetrance and late onset of phenotypes preclude efficacy in a high-throughput screen.

ENU is expected to induce mutations randomly, but large genes are likely to have more mutations than small ones. *Ttn* is mutated in nearly every ENU screen because of its size (the cDNA is 81,843 base pairs, encompassing 192 exons), and 20 alleles were isolated here. Therefore, we cannot be certain that some genes had mutations because of their size, rather than their ability to modify *Mecp2*. *Tet1* had three alleles, and is a relatively small gene, which has 13 exons. *Birc6* also had three alleles, yet has 73 exons. Therefore, although alleles are called, some of the lesions may not suppress phenotypes. This scenario shows that mating all lines for inheritance and segregation is ideal. However, all of the founder lines reported here were not mated owing to time, cost, and animal space limitations. Our hope was that by identifying pathways, the number of animals used in breeding could be reduced. Candidate genes in any line can be confirmed after reanimation by IVF or by using CRISPR/Cas9 genome editing.

All of the potential modifiers were not identified. First, the screening relied on subjective health parameters and did not include a quantitative test for neurological function. Thus, modifier traits could be overlooked and/or misjudged. Although this may imply a need for more prescriptive assessments of RTT-like phenotypes, time and cost preclude such evaluations in a high-throughput screen. DNA variants could also be missed; as evidence, line 856 had a strong LOD score for a second modifying locus on Chromosome 6, yet WES did not identify a candidate in the region. Many factors can influence the observation that WES falls short of identifying all lesions, in part because DNA quality can influence depth of coverage, making heterozygous mutations difficult to call. Moreover, genome annotation in the mouse remains incomplete, with missing segments and incompletely annotated exons. For example, the suppressor in line 1395 mapped to an unannotated region of mouse Chromosome 7. Finally, loci present in the two inbred strains used here may also influence the penetrance of traits, yet modifying loci in the strain backgrounds were not a priority for identification.

The inheritance of haplotypes can also make linked ENU-induced mutations difficult to sort as causative (Neuhaus and Beier 1998). In line A_333N, two mutations had very strong association scores as potential modifiers: A mutation in SH3 and multiple ankyrin repeat domains 1 (*Shank1*) was a strong candidate gene based on its autism associations, whereas a mutation in *Fan1* would be consistent with the DDR pathway. However, these two genes lie only 20 Mb apart on Chromosome 7. Nine animals showed crossovers between the two loci, suggesting that the mutation in *Fan1* was the more strongly supported modifier, and there was no association with their inheritance together. In contrast, although *Aacs* and *Atp8a1* lie 60 Mb from each other on Chromosome 5, the mutations in each showed strong association with trait improvement, and their inheritance together further improved the traits. These data show that in these small family data sets without additional fine structure mapping crosses, candidate genes could be resolved by recombination and association analysis. However, the associative candidate genes are not confirmed by fine mapping, complementation, or functional studies, so should be confirmed by additional experiments.

An unbiased modifier approach has implicated several pathways in RTT pathogenesis, one of which supports the idea of improving synaptic signaling for treatment, as expected from published evidence. Even so, our study suggests additional avenues for intervention. For example, CD22 responds to 2-hydroxypropyl-beta-cyclodextrin, which is in phase 2 and 3 clinical trials for Niemann–Pick disease, to reduce microglia-associated defects (Cougnoux et al. 2018), and antibodies directed against CD22 reduce microglial impairment in aging brains (Pluvinage et al. 2019). In addition, other pathways suggest that alternative avenues for intervention should be considered when studying RTT pathogenesis. Lipid homeostasis is directly regulated by an interaction between MECP2 and the NCOR1 corepressor complex (Kyle et al. 2016). Mutations in *MECP2's* NCOR interaction domain (NID) cause classical RTT in humans (Heckman et al. 2014) and RTT-like phenotypes in mice (Lyst et al. 2013), highlighting the importance of the NCOR1 interaction with MECP2 in RTT pathology. The NCOR complex is a master regulator of metabolism, playing roles in lipid biogenesis, glucose utilization, and mitochondrial energy efficiency (Mottis et al. 2013). It follows that metabolism, which is a highly druggable target, should be carefully examined in *Mecp2* mice and in RTT patients.

This work suggests a role for DDR in RTT pathology for the first time. The genomic DNA of all cells must be protected from detrimental changes (Madabhushi et al. 2014), and MECP2's absence is associated with the accumulation of genetic damage (Muotri et al. 2010; Squillaro et al. 2010; Alessio et al. 2018). DSBs occur in all neural precursor cells and are important for normal neuronal development. However, the role of DNA integrity in mature neurons is less thoroughly understood. In neurons, topoisomerase II-mediated DSBs occur to relieve topological stress during transcription (Suberbielle et al. 2013; Madabhushi et al. 2015). This in turn impacts gene expression, especially of neuronal early response genes important for synapse development and maturation, neurite outgrowth, the balance between excitatory and inhibitory synapses, and learning and memory. Robust mechanisms must be in place to rapidly and efficiently repair these DSBs; when these mechanisms fail, the accumulation of DSBs in the brain is a contributor to number of neurological diseases (Frappart and McKinnon 2008; Merlo et al. 2016). It is likely that elevated RBBP8 in *Mecp2-*null cells is a result of increased DSBs, making increased HDR and/or decreased NHEJ in *Mecp2-*null neurons important for understanding RTT pathology.

Altogether, the *Mecp2* suppressor mutations paint a picture of altered metabolism and DNA damage that modulate synaptic function to cause pathology in Rett syndrome. *FAN1* is also a modifier of Huntington's disease phenotypes in humans (Genetic Modifiers of Huntington's Disease (GeM-HD) Consortium 2019); thus, the modifiers found here could be common to other neurological diseases. Many of the lines with the largest degree of symptom improvement carry at least two modifiers, which may reflect a lack of quantitative phenotyping. Even so, combining modifiers often improves symptoms, suggesting that combination therapies for RTT will be more effective than any single therapy. The results underscore the power of a genetic screen for understanding RTT biology because they demonstrate how a modifier screen in mammals is possible, especially for disease genes that are not present in more tractable genetic organisms. With new sequencing technologies and statistical approaches, such a screen should be amenable for nearly any gene for which phenotypes can be clearly assessed. Modifier screens in model organisms may thus help to identify the multitude of genetic variants that influence human disease presentation, as they may point to therapeutic entry points (Enikanolaiye and Justice 2019).

## Methods

### Animals

All animal experiments were conducted under protocols approved by local Animal Care and Use Committees at Baylor College of Medicine (BCM) or at The Centre for Phenogenomics (TCP) accredited by the American Association for Laboratory Animal Care (AALAC) and Canadian Council on Animal Care (CCAC), respectively. Congenic 129.*Mecp2^tm1.1Bird^*^/+^ female mice were maintained by backcrossing females to males of the 129S6/SvEvTac strain. 129.*Mecp2^tm1.1Bird^*/Y (*Mecp2*/Y, which are *Mecp2*-null) and age matched wild type (+/Y) littermate controls were housed in plastic Tecniplast cages with corncob bedding in rooms alternating 12-h and 12-h periods of light and dark, were provided acidified water and a Harlan Teklad 2920X diet ad libitum (19.1% protein, 6.5% fat; 0% cholesterol) (BCM) or Harlan Teklad 2919 (19% protein, 9% fat) (The Center for Phenogenomics-TCP). The BCM colony allowed helicobacter, whereas mice were embryo rederived into TCP, which allows no common pathogens. C57BL/6J males were obtained from The Jackson Laboratory (Bar Harbor, ME) at six weeks of age, and injected with three weekly doses of 100 mg/kg ENU at 8 wk as described (Kile et al. 2003). After recovery of fertility, ENU-treated males were mated to 129.*Mecp2^tm1.1Bird/+^* females, and their N1 male offspring were genotyped for the *Mecp2* mutation according to The Jackson Laboratory standard protocol using an Applied Biosystems thermocycler and resolution on a QiAxcel (Qiagen).

For 30 of the founder lines, N1 males showing signs of improvement were mated to 129S6/SvEvTac females, or sperm was frozen and IVF was performed with oocytes from 129S6/SvEvTac females by the Cryopreservation and Recovery Core, TCP.

### Sequencing pipeline

DNA was extracted from mouse tails using standard methods (Supplemental Methods). For sequencing by The Centre for Applied Genomics (TCAG), 500 ng of genomic DNA was fragmented to 200 bp on average using a Covaris LE220 instrument. Sheared DNA was end-repaired and the 3′ ends adenylated before ligation of adapters with overhang-T. PCR amplified genomic libraries were exome captured, pooled, and sequenced with the TruSeq SBS sequencing chemistry using a V4 high-throughput flowcell on a HiSeq 2500 platform following Illumina's recommended protocol. Approximately 6–8 gb of raw paired end data of 126 bases were generated per exome library. Inbred strain polymorphisms as well as systematic sequencing artifacts, were removed from consideration if identified in the parental strains, dbSNP, or other founder males sequenced in this study, and detrimental variants were called using standard tools and custom scripts (Supplemental Code) (Li et al. 2009; Li and Durbin 2010). Candidate lesions were confirmed by Sanger sequencing before genotyping N3 animals (Supplemental Fig. S3). Primer sequences for each locus are in Supplemental Table S4.

### Quantitative reverse transcription polymerase chain reaction (qRT-PCR)

Whole brain tissue was homogenized and total RNA isolated (Qiagen RNeasy Lipid Tissue Mini Kit) for qRT-PCR analysis (Kyle et al. 2016). Expression was normalized to TATA-binding protein (*Tbp*) as an internal control and results analyzed using the 2^−(ΔΔCT)^ method. Primer sequences are in Supplemental Table S5.

### Statistics

Complex versus Mendelian inheritance was assessed from the number of animals that inherited trait improvement or longevity in each N2 family (Supplemental Table S2). For each line, the decomposition of N3 improved cases across N2 families is tested for the Mendelian hypothesis by generating data conditional on whether or not N2 animals inherited the trait given the presence (with probability 0.50) or absence (with probability 0.25) of an N3 offspring with improvement in the family. The *P*-value is calculated considering the number of as-or-more extreme cases under the same family configuration than the one observed.

In screen 2, associations of genetic loci with outcomes based on subjective health parameters were assessed using cumulative link models (CLM) suitable for ordinal data (Agresti 2011; https://cran.r-project.org). The health score, limb clasping, tone, activity, and tremor scores were fitted to separate models. Association with body weight at 8 wk was inferred using linear models. In either model, random effects were included to account for potential clustering of outcomes within families. However, in almost all cases, random effects had a negligible variance suggesting that there was no significant clustering of the outcomes, allowing for the fitted models to include only fixed effects. The associations of genetic loci with the longevity (time to sacrifice) were assessed using parametric survival models, in which the final model for each line was selected using the Akaike Information Criterion (AIC) (Akaike 1974). For each line, marginal effects of each gene were investigated, as well as pairwise gene–gene interactions on each trait and the survival models. All association analyses were performed using the R statistical programing software (R Core Team 2019), along with the packages ordinal (https://cran.r-project.org), lme4 (Bates et al. 2015), and flexsurv (Jackson 2016).

Kaplan-Meier survival curves in Figures 2B and 4B were generated in GraphPad Prism 8 followed by the Mantel-Cox test (log-rank comparison). Comparisons between two groups, as in qPCR experiments, were carried out in GraphPad Prism 8 using the two-sample Student's *t*-test.

## Data access

The N1 founder whole-exome sequencing data generated in this study have been submitted to the NCBI BioProject database (https://www.ncbi.nlm.nih.gov/bioproject/) under accession number PRJNA603006.

## Competing interest statement

The authors declare no competing interests.

## Supplementary Material

Supplemental Material
