# Supplementary material for: Suppressor mutations in Mecp2-null mice implicate the DNA damage response in Rett syndrome pathology
Source: Genome Res. 2020 Apr;30(4):540–52. doi: 10.1101/gr.258400.119 (PMC7197480; doi:10.1101/gr.258400.119)
Supplement: Supplemental Material [file supp_30_4_540__index.html]

Suppressor mutations in Mecp2-null mice implicate the DNA damage response in Rett syndrome pathology — Supplemental Material 

# Suppressor mutations in *Mecp2*-null mice implicate the DNA damage response in Rett syndrome pathology

## Supplemental Material

- Supplemental\_Table\_S1.xlsx
- Supplemental\_Table\_S2.xlsx
- Supplemental\_Material.pdf
