# Supplementary material for: Suppressor mutations in Mecp2-null mice implicate the DNA damage response in Rett syndrome pathology
Source: Genome Res. 2020 Apr;30(4):540–52. doi: 10.1101/gr.258400.119 (PMC7197480; doi:10.1101/gr.258400.119)
Supplement: Supplemental Material [file supp_gr.258400.119_Supplemental_Material.pdf]

Table of Contents for Supplemental Material

Supplemental Table_S1 is provided as an .xlsx file.

Supplemental_Table_S2 is provided as an .xlsx file.

File	*Page*
Supplemental_Methods.pdf	2
Supplemental_Code.pdf	3-5
Supplemental_Table_S3.pdf: Association test p-values	6
Supplemental_Table_S4.pdf: Primer sequences for Sanger sequencing	7-8
Supplemental_Table_S5.pdf: Primer sequences for qRT-PCR	9
Supplemental_Fig_S1.pdf: Lesions in *Tet1* and *Birc6* fall into conserved protein domains	10
Supplemental_Fig_S2.pdf: Lesions in *Mre11a*, *Brca1*, *Rad50*, *Brca2*, *Fan1* and *Spin1* fall into conserved protein domains	11
Supplemental_Fig_S3.pdf: Sanger sequence traces	12-17

Supplemental methods

Mouse DNA: To obtain DNA, mouse tails were digested overnight at 55ºC in tissue lysis buffer containing proteinase K, followed by purification with phenol chloroform, and precipitation with isopropanol. Resuspended DNA pellets were quantified using both a Nanodrop (Thermo Fisher) and Qubit (Thermo Fisher).

Sequence analysis: Genomic libraries were amplified by PCR using 10 cycles and 750 ng of the PCR-enriched library was hybridized with biotinylated probes that target exonic regions; the captured exome libraries were amplified by an additional 10 cycles of PCR. Exomic libraries were validated on a Bioanalyzer 2100 DNA High Sensitivity chip (Agilent Technologies) for size and by qPCR using the Kapa Library Quantification Illumina/ABI Prism Kit protocol (KAPA Biosystems) for quantities.

Sequence reads were mapped to the mm10 reference genome with *bwa*, and variants were called with *samtools* requiring a minimum SNP quality of 20 and a read depth $\geq$ 5. Putative Insertion-Deletion (InDel) calls were realigned and SNP quality was recalibrated with GATK. Custom scripts combined these programs to annotate variants with respect to their predicted impact on protein sequence (Supplemental_Code).

Supplemental_Code.pdf

Custom scripts for calling detrimental variants:

==== project <Suppressor mutations screening in Mecp2-null mice>====
==== operating system and method <Linux clust and Linux commands see below>====
==== copyright 2020 Rong Zeng (rong.zeng@uhn.ca)====

include <mouse reference genome:GRCm38 or mm10>

include <69 Mouse lines:
520,654,751,4799,918_15L,J_57L,A_27R,A_333N,M_199LL,M_333R,136,137,591,722,933_15N,A_87N,A_120N,A_134L,A_83L,620,A_189N,A_173N,M_420L,M_79N,920_39R,S_165N,4633,J_136N,J_157N,A_230RR,604,A_347N,272,747,752,4604,4730,904_16LL,905_20N,920_36LL,929_11R,934-2N,J_21N,A_252LL,A_315N,A_322N,A_345L,A_210N,F_227N,F_245L,F_251L,J_3N,J_92N,J_129L,M_351LL,M_453N,M_466LL,S_23RR,S_36R,S_158R,S_239L,S_301N,S_305L,U_45N,U_372R,U_672N,U_734LL,F_99N,F_311L>

include Wellcome Trust Sanger Institute mouse SNP database <Newsnp.vcf>

include <NGS tools: Fastx_toolkits,Fastqc,Trimmomatic,BWA,SAMtools,Picard,GATK,Annovar>

include <script.js> adapted from the script by Pierre Lindenbaum at "L'Insitut du Thorax"

```
//  function accept(ctx)

   {
   var y,g2;
   var sampleList=header.getSampleNamesInOrder();

   var g1=ctx.getGenotype("LINE");
   /** ignore non-called */
   if(g1== null || ! g1.isCalled() ) return false;
   /** loop over the other samples */
   for(y=0;y< sampleList.size();++y)
      {
      g2=ctx.getGenotype( sampleList.get(y) );

      if(g2.getSampleName().equals(g1.getSampleName())) continue;
      /** ignore non-called */
      if(! g2.isCalled() ) continue;

      /** is g1==g2 ? */
      if( g1.sameGenotype( g2 ) ) return false;
      }
   /* found no other same genotype */
   return true;
   }
   accept(variant);
```

====LINUX commonands for next generation sequencing data analysis, variant calling and filtering in ENU Mecp2/y mice project====

```
# reads alignmnet <Forward reads>
bwa aln genome.fa ./LINE/LINE_R1.fastq.gz > LINE_R1.sai &

# reads alignmnet <Reverse reads>
bwa aln genome.fa ./LINE/LINE_R2.fastq.gz > LINE_R2.sai &

# SAM format <paired-end reads>
bwa sampe -f LINE.sam -r "@RG\tID:LINE\tLB:LINE\tSM:LINE\tPL:ILLUMINA" genome.fa LINE_R1.sai LINE_R2.sai ./LINE/LINE_R1.fastq.gz ./LINE/LINE_R2.fastq.gz &

# sort the SAM file and convert to BAM file

java -jar SortSam.jar SO=coordinate INPUT=LINE.sam OUTPUT=LINE.bam VALIDATION_STRINGENCY=LENIENT CREATE_INDEX=true &


# Check the head of LINE.bam
samtools view -H LINE.bam

# flag possible PCR duplicates
java -Xmx25G -jar MarkDuplicates.jar INPUT=LINE.bam OUTPUT=LINE.marked.bam METRICS_FILE=metrics CREATE_INDEX=true VALIDATION_STRINGENCY=LENIENT &

# creates a table of possible indels
java -jar GenomeAnalysisTK.jar -T RealignerTargetCreator -R genome.fa -o LINE.marked.bam.list -I LINE.marked.bam &

# realigns reads around possible indels
java -jar GenomeAnalysisTK.jar -I LINE.marked.bam -R genome.fa -T IndelRealigner -targetIntervals LINE.marked.bam.list -o LINE.marked.realigned.bam &

# fixing the mate information
java -jar FixMateInformation.jar INPUT=LINE.marked.realigned.bam OUTPUT=LINE.fixed.bam SO=coordinate VALIDATION_STRINGENCY=LENIENT CREATE_INDEX=true &

# base quality Recalibration
java -jar GenomeAnalysisTK.jar -T BaseRecalibrator -I LINE.fixed.bam -R genome.fa -knownSites SNPMOUSENEW.vcf -o recalibration_reportLINE.grp &

# printReads
java -jar GenomeAnalysisTK.jar -T PrintReads -R genome.fa -I LINE.fixed.bam -BQSR recalibration_reportLINE.grp -o outputLINE.bam &


# Unified Genotypes from all lines < outputLINEs.bam has included outputline.bams from all founder mouse lines in the study>
java -jar GenomeAnalysisTK.jar -glm BOTH -R genome.fa -T UnifiedGenotyper -I outputLINEs.bam --dbsnp SNPMOUSENEW.vcf -o thirtyonesnp.vcf -metrics snps.metrics -stand_call_conf 50.0 -stand_emit_conf 10.0 -dcov 200 -A AlleleBalance &
```

```
# change specific LINE name in the script.js and filter variants of specific line comparing with all lines
java -Xmx50g -jar jvarkit/2014.10.15/dist/vcffilterjs.jar -f script.js ALLLINEsnp.vcf > LINE.vcf&

# splitting the genotypes of specific lines
java -Xmx25g -jar GenomeAnalysisTK.jar -R genome.fa -T SelectVariants --variant LINE.vcf -o
LINE.vcf -sn LINE &

# filter variants of specific line comparing with variants in the
java -Xmx25g -jar GenomeAnalysisTK.jar -R genome.fa -T SelectVariants --variant LINE.vcf --
discordance Newsnp.vcf -o LINEv.vcf &

# extract heterozygotes
java -Xmx4g -jar GenomeAnalysisTK.jar -T SelectVariants -R genome.fa --variant LINEv.vcf -select
'vc.getGenotype("LINE").isHet()' -o LINEhet.vcf &



# hard-Filtering
java -Xmx4g -jar GenomeAnalysisTK.jar -R genome.fa -T VariantFiltration -o LINEf.vcf --variant
LINEh.vcf --clusterWindowSize 10 --filterExpression "MQ0 >= 4 && ((MQ0 / (1.0 * DP)) > 0.1)" --
filterName "HARD_TO_VALIDATE" --filterExpression "DP < 5 " --filterName "LowCoverage" --
filterExpression "QUAL < 30.0 " --filterName "VeryLowQual" --filterExpression "QUAL > 30.0 && QUAL
< 50.0 " --filterName "LowQual" --filterExpression "QD < 1.5 " --filterName "LowQD" &

# convert the VCF file to the annovar file format:
annovar/convert2annovar.pl --format vcf4old --includeinfo LINEf.vcf > LINEt.annovar &

# annotate variant
annovar/annotate_variation.pl --buildver mm10 LINEt.annovar mm10db/ &

# convert annotated file to .txt file <exonic_region>
annovar/table_annovar.pl -otherinfo -buildver mm10 LINEt.annovar.exonic_variant_function mm10db/
-protocol refGene -operation g -nastring NA &

# convert annotated file to .txt file <exonic_intronic boundary region>
annovar/table_annovar.pl -otherinfo -buildver mm10 LINEt.annovar.variant_function  mm10db/ -
protocol refGene -operation g -nastring NA &
```

Supplemental_Table_S3: Association test p-values for marginal gene effects and gene-gene interactions

**Line**	**Gene**	**Longevity**	**Health**	**Limb Clasping**	**Activity**	**Muscle Tone**	**Weight**
352	***Prdm15***	**< 0.001**	-	-	-	-	-
856	***Zzz3***	**< 0.001**	-	-	-	-	-
895	*Sqle*	0.330	-	-	-	-	-
	Rbbp8	**< 0.001**	-	-	-	-	-
	Sqle*Rbbp8	**< 0.001**	-	-	-	-	-
520	*Mark4*	0.580	0.138	0.191	0.365	0.840	-
	Cd22	0.960	**0.037**	**0.015**	0.325	0.646	-
	Clint1	0.270	0.498	0.212	0.663	0.755	-
	Fnip1	0.780	0.970	0.480	0.766	0.956	-
	Kdm4a	**0.035**	**0.024**	**0.023**	0.344	0.866	-
	*Mark4 * Cd22*	nc	nc	nc	nc	nc	-
	*Mark4 * Clint1*	0.129	0.714	0.419	0.048	0.116	-
	*Mark4 * Fnip1*	0.900	0.572	0.180	0.453	0.396	-
	*Mark4 * Kdm4a*	0.338	0.040	0.200	0.219	0.413	-
	Cd22 * Clint1	**0.042**	0.344	0.694	**0.048**	**0.031**	-
	*Cd22 * Fnip1*	0.830	0.383	0.725	0.295	0.140	-
	*Cd22 * Kdm4a*	0.448	0.313	0.593	0.985	0.658	-
	*Clint1 * Fnip1*	0.328	0.434	nc	0.902	0.652	-
	*Clint1 * Kdm4a*	0.090	0.471	0.754	0.057	0.134	-
	*Fnip1 * Kdm4a*	0.310	0.193	0.927	0.076	0.066	-
4654	***Gtf3c5***	**0.030**	0.974	0.055	**0.010**	**0.014**	0.460
	Dennd4a	0.170	0.549	0.273	0.132	0.326	0.313
	Tm7sf2	0.096	0.267	**<0.001**	**<0.001**	**<0.001**	0.251
	*Gtf3c5 * Dennd4a*	0.492	0.105	0.535	0.109	0.858	0.351
	*Gtf3c5 * Tm7sf2*	0.065	0.742	0.844	0.189	0.110	0.338
	Dennd4a * Tm7sf2	**0.030**	0.086	0.298	0.205	0.472	0.822
4751	*Paox*	0.240	0.495	0.222	0.134	0.199	-
	Argef15	**<0.001**	0.840	0.263	**<0.001**	**<0.001**	-
	Spin1	**0.018**	0.322	**0.004**	0.321	0.079	-
	*Paox * Argef15*	0.133	0.525	0.993	0.786	0.516	-
	*Paox * Spin1*	0.921	0.058	0.807	0.669	0.948	-
	*Argef15 * Spin1*	0.909	0.415	0.123	0.278	0.100	-
4799	***Atp8a1***	0.170	**0.004**	**0.003**	**0.007**	**0.005**	0.552
	Aacs	**0.011**	**< 0.001**	**< 0.001**	nc	nc	0.556
	Atp8a1 * Aacs	**0.025**	**0.006**	0.191	nc	nc	0.104
918_15L	***Celsr3***	**0.002**	**0.002**	**< 0.001**	nc	nc	0.332
J_57L	*Col24a1*	0.990	0.234	0.135	0.655	0.960	0.424
	Apoa5	0.059	0.769	**0.001**	**0.019**	**0.002**	0.061
	Hcn2	**0.022**	0.276	0.959	**< 0.001**	**< 0.001**	**0.041**
	*Col24a1 * Apoa5*	0.190	0.706	0.622	0.703	0.202	0.007
	*Col24a1 * Hcn2*	0.180	0.392	0.117	0.779	0.736	0.583
	Apoa5 * Hcn2	0.555	0.433	0.149	0.058	**0.043**	0.169
A_27R	***Tet1***	**< 0.001**	0.460	**< 0.001**	**< 0.001**	**< 0.001**	-
	Pelo	0.950	0.337	0.132	0.691	0.968	-
	*Tet1 * Pelo*	0.394	0.553	0.735	0.764	0.668	-
A_333N	***Shank1***	0.071	**0.035**	**< 0.001**	-	-	-
	Fan1	**0.004**	**0.011**	**< 0.001**	-	-	-
	*Shank1 * Fan1*	0.782	0.730	nc	-	-	-
M_199LL	***Birc6***	0.320	**< 0.001**	**-**	**-**	**-**	**< 0.001**
	Dbnl	0.082	0.662	-	-	-	0.928
	Birc6 * Dbnl	**< 0.001**	0.709	-	-	-	0.675
M_333R	***Zbtb41***	**0.017**	0.887	-	0.117	0.114	-
	Mtor	0.430	0.340	-	0.644	0.433	-
	*Zbtb41 * Mtor*	0.939	0.106	-	0.861	0.728	-

nc: model failed to converge; - : phenotype not tested for association; The significant associations identified at the 5% significance level are in BOLD.

Supplemental_Table_S4: Primers used for Sanger Sequencing

**Line (Gene)**	**Forward Primer (5' to 3')**	**5' to 3' Reverse Primer**
352 *(Prdm15)*	GGACAGGGCAAATTAAGCAA	CTCCTTCGACCACCATCAGT
856 *(Zzz3)*	GCCAGAGCTGTCCTCTATCCT	TGCTGACTGGCACTCTCT
895 *(Sqle)*	CTTGTATAAGGAGGTGCTTAATGGA	GAAATCCACCTGCCTCTGC
895 *(Rbbp8)*	CATTCTCTCTTGACAAGGTGACAG	TTTCTTTGTGGTGTCAGGCAGTTC
520 *(Cd22)*	TGCTGGGAAAGCTCATGTGTA	CCTCTTACCATAGGCCGCTT
520 *(Kdm4a)*	TTGAAAGGTACTGCACCCCG	CCAAGCACACAGTAGGCACT
520 *(Clint1)*	TGACAAGTCAGAAAAACCTGAAGTC	CTCGCTCCGTTACAACCTTT
520 *(Mark4)*	CTGTGGGTGGTGGCTTTTTG	AAGTTCCCGTGGCTGTACC
520 *(Fnip1)*	ACTGCAGTGTGTAATGCACC	ACAACAGAAAGCAGTTCCAAGG
4654 *(Tm7sf2)*	AAGTCAAGGCAGAGTCGCAA	GCAGGCTGTAGGTGAATGGT
4654 *(Gft3c5)*	GCCAACCCCTTAGACTTCCA	CCCATTAGTGCTGTTGCCCA
4654 *(Dennd4a)*	AGTGCAGCAGAGACCAAGAGA	CTACAGAGCTGGCACTCACAC
4751 *(Arhgef15)*	GCACCTCCATCTTCTGGGAG	GAGCTGGTTCTGGGGAGATG
4751 *(Spin1)*	AGGCCAAGCCCTCTGTCTAT	GTTCGCAGGGACATCTAAGC
4751 *(Paox)*	CTCTGGAGGACCCTGAGCTA	AAGGAGCGGTTACCGAAGC
4799 *(Aacs)*	ATCCGTGGATACCAGGCAAG	CCAGGTCTCCCCCTAGACAT
4799 *(Atp8a1)*	ACTTGGAGCTACAGCCATTGA	CGAAACCACTTGTCTCCAGC
918-15L *(Celsr3)*	TGTCATGAACTCTCCGGTGG	GCAGTCCACATACCATGCTG
J_57L *(Hcn2)*	TTCCTGCAGCGCCAATTC	CCGGTGACTCGAACTGC
J_57L *(Apoa5)*	AATAAGGCCCTGAGCAGACT	ATGAGTACCAGAAGCTGGGT
J_57L *(Col24a1)*	ACAAGGGCATTGGATATCTTGA	GGCACTTACCTTTAAGCCTTCT
A_27R *(Tet1)*	CTGTCAGACATGGGGCATCA	AGTCGCCAGCTAAGAGAGGT
A_27R *(Pelo)*	GTGAACATCCCCAGGAAACG	GGCTCCTCCCCATTCTAAAAC
A_333N *(Fan1)*	TGCCAGTGTGGCTCACATAC	GCTAGGATGCAGGAAGCACT
A_333N *(Shank1)*	ATGAGGTCTAACCAGGCCCA	AGTTGGAGTGGAGAGGGCTT
M_199LL *(Birc6)*	TGCTTGGTGACTGACAGTGTA	TGCGAGGTGAGGTTGAATGT
M_199LL *(Dbnl)*	TCACTGGGGCAAGTGGTTCT	TCAAGGATTTCACCAAGTAGCCA
M_333R *(Zbtb41)*	TGCAATGCGAAGTTTCCGTC	ATGAACGAGCTGCCATTTGC
M_333R *(Mtor)*	ACTGCTGTGCTCTATGACCTG	TCCGAGTTTCTGCCTCTGAT
J_157N *(Tbl1xr1)*	TGCAAGCAAAACTCCAGTGC	CCATGGGACATAAAGCACCC
S_165N *(NCoR1)*	ATGCAGGCCTGGTAGAACAT	AAAGCTAAAGGTCAGGTCTCTC
591 *(Tet1)*	GCAGCCGAGAATGAAGTCCA	GCGGCGTAGAATTACATGGG
591 *(Arhgap15)*	CAGAAATGAACCGAACCCCC	CCAGAACAGGCTGCAAAACAA
591 *(Anapc5)*	TGGCTGTCAGTCTTCCTTCT	TCTTTCCCGCCCATTACCG
591 *(Mmadhc)*	CATTTCTCCTTTCCTCCAGTTTTT	AGTGCCAGCTGCTAGTTTCC
591 *(Hat1)*	TGTGTGATACCTGTTGGTTTTGTC	ATGGAGGAGGGTTCCAAAAGG
137 *(Atp6v1b1)*	CAGAGGTTCTCACGCTCCTT	GGACTGATTTAGAGCGCCGT
137 *(Grm8)*	GGAGAAAGAGGGGTGCCTTG	GGGTGGGTCTCCATTAGCAC
722 *(Bscl2)*	CTGGAAGCACACTCAGTGGA	CAGAGGCAGACCTAGAACCC
722 *(Kif21a)*	TCCACGCCATGAACAAAGAG	CTGTTCCAAGACATCCAGCCC

722 *(Adcy6)*	GACCTGTGCCTCTGTCCTTTT	TGATGTAGACGAAGAACACGGG
722 *(Map3k12)*	GCAGGATGAGACAGGTACAGAG	TTTTGTCAGTCCAGTCTCCCA
933-15N *(Foxn4)*	TGGAACAGGTTTCTGCCTCC	TATATGGACATGCCCCCAGAA
933-15N *(Parp1)*	TCAGCCCCGGTGTCTGTAAT	GGAAATTTGGGAGCTCACGG
933-15N *(Cox16)*	TGCGCTGAGTCTACAACTTC	CCCTAGTCAGCAGTATCCGA
933-15N *(Tdp1)*	ACAGTGCGAATCTGTCCAAG	GGATTGGAATCCTCACCACG
A87N *(Psmb2)*	AGCGATTTGGGTGAAGACTGT	GCTTTCTGCTGTTGAAGGCTG
A87N *(Emcl)*	CTTCCCAGAGTCTGAACCCC	CAGTTCAAGGACCTACAGGCA
A87N *(CD37)*	TTGTGAGTCTACTCATGGCTACC	AGCCCTACCTGGGAAGATAGTA
A87N *(Pcf11)*	ACGCCTGTCGGGATTATCAG	CCAAAGACCAAGCCCGAGT
A87N *(Cep72)*	CTAGCTCAGGCCAAGCATGT	ACTTGTCTTCCCCTTGTCCC
A120N *(2310022B05Rik)*	AGGTAAATTGAGGTCGGGGC	ACGGGGAAAACCTGGCAAC
A120N *(Unc79)*	TCCAATCTGTCAATTTCTGAGGC	CACCACTCTCCTGTCTCCTGA
A120N *(Eif2b5)*	GGTCTTTCTGGTCTTAGGGCA	GGTACTGGACTCTGGACAACTT
A120N *(Fanca)*	GAGCATGCTGGGTAAGAGACT	TAACCGGAACGTCCGTCAC
A120N *(Sidt2)*	TTGTCAAGGTGACCTCCAAGAA	TTTCTCAGGCAGCACAAGGT
A134L *(Scn11a)*	ACTTGCTGGGGAAATCCACT	GCCAGCGTGCATACTTATCTT
A134L *(Gm14496)*	TGGCTTCTTGTGCAGGGTTG	TAGGCTGGACTCGAACTCAGAA

Supplemental_Table_S5: Primers Used for qRT-PCR

Gene	Forward Primer (5' to 3')	Reverse Primer (5' to 3')
Tbp	CCTTGTACCCTTCACCAATGAC	ACAGCCAAGATTCACGGTAGA
Rbbp8	AATGGTCAACAGGATCAAGTAGC	GTAGCCGGTTAATGCCAGAAAA
Mre11a	CCGAGAAAGCAGACAGAGAAA	AAAGGCTGAGGTGGAGGTCT
Rad50	GGTGAACAAGGACCTCGACA	CCGCCAAAGATCACGAATAA
Nbn	CCCATGTGGAAGCTGCTC	AGTTTTTCCTCCCAACAACG
Atr	GAATGGGTGAACAATACTGCTGG	TTTGGTAGCATACACTGGCGA
Cdk1	GACAGAGAGGGTCCGTCGTA	TGGCCAGTGACTCTGTGTCT
Rpa2	ATGGATGTTCGTCAGTGGGT	AAAGGCCACCAAGCTCTTTT
Xrcc5	AGATTGCCAGCTTTGAGGAA	GCTTTGATGCAGTCCATACTTTT
Trp53bp1	TGGAGTCTTCTCATTTGGGT	GCAGCTTCTACTGTCACTGCC
Prkdc	AAGGATGTATGCTGCCTTGG	ACCACCATTCCCAAAGCTC

Supplemental_Fig_S1.pdf

A

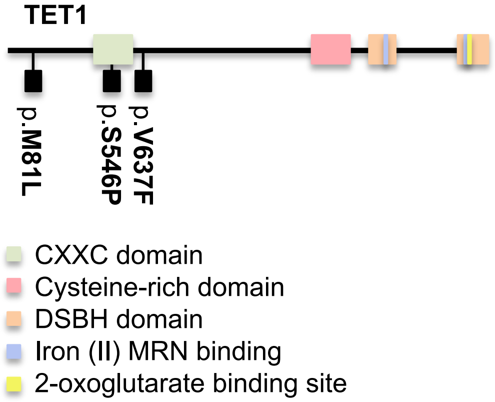

	p.**M81L**	p.**S546P**	p.**V637F**
RAT	-TRAGAARLNQDRNQV-	-EGGISHV**S**MTSSADD-	-EKKPK**V**LKTDFNN-
MOUSE	-TRAGAAR**M**NRDRNQV-	-EGEISRV**S**ITGSA-D-	-EKKPK**V**LKTDFNN-
HORSE	-TRAGAAR**M**NLDRTEV-	-EEESSQI**S**KTSMVD--	-EKKPK**V**LKADFDN-
COW	-TRAGAAR**M**NLDRTEV-	-EGGSSQI**S**ATRLVD--	-EKKPK**V**LKADFDN-
CAT	-TRAGAAR**M**NLDRTEV-	-EGGSSQI**S**VTSMVD--	-EKKPK**V**LKANFDN-
DOG	-TRAGAAR**M**NLDRTEV-	-EGESSQV**S**MTSVVD--	-EKKPK**V**LKADFDN-
HUMAN	-TRAGAAR**M**NLDRTEV-	-DRGSSQV**S**VTSTVHV-	-EKKPK**V**LKADFDN-
CHIMP	-TRAGAAR**M**NLDRTEV-	-DRGSSQV**S**VTSTVHV-	-EKKPK**V**LKADFEN-

B

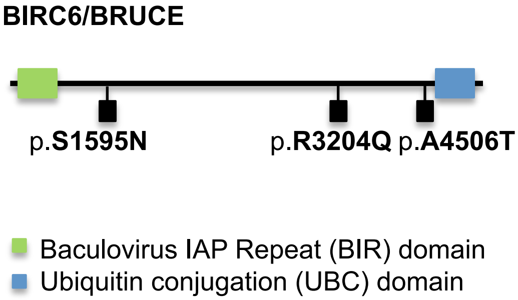

	p.**S1595N**	p.**R3204Q**	p.**A4506T**
XENOPUS	-DDASTFTV**S**TFGVTP-	-LQATPPH**R**RARSAAW---	-TAEIVS**A**ATTSLRQ-
RAT	-DDASTFTV**S**SFGVTP-	-LQATPPH**R**RARSAAW---	
MOUSE	-DDASTFTV**S**SFGVPP-	-LQATPPH**R**RARSAAW---	-TAEIVH**A**ATANLRQ-
HORSE	-DDA----M**S**SFGVTP-	-LQATPPH**R**RARSAAW---	-TAEIVY**A**ATTSLRQ-
COW	-DDA----M**S**SFGVTP-	-LQATPPH**R**RARSAAW---	-TAEIVY**A**ATTSLRQ-
CAT	-DDASTFTV**S**SFGVTP-	-LQATPPH**R**RARSAAW---	-TAEIVH**A**ATTSLRQ-
DOG	-DDA----M**S**SFGVTP-	-LQATPPH**R**RARSAAW---	-TAEIVH**A**ATTSLRQ-
HUMAN	-DDA----M**S**SFGVTP-	-LQATPPH**R**RARSAAW---	-TAEIVY**A**ATTSLRQ-
CHIMP	-DDA----M**S**SFGVTP-	-LQATPPH**R**RARSAAW---	-TAEIVY**A**ATTSLRQ-

Supplemental_Fig_S1_Legend: Amino acid alignments generated using Clustal Omega show that the individual amino acids as well as their surrounding sequence is well conserved among organisms.
(A): Protein structure of TET1: the missense mutations cluster around the CpG binding domain.
(B) Protein structure of BIRC6: The mutations occur throughout the protein, but not in known functional elements.

Supplemental_Fig_S2.pdf

A MRE11

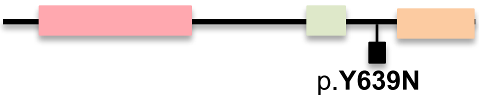

Phosphoesterase domain
DNA Binding A
DNA Binding B

p.**Y639N**	
XENOPUS	–RNMPKKT**Y**SEDIEDD–
RAT	–RNVATKN**Y**SETIEVD–
MOUSE	–RNVAPKN**Y**SETIEVD–
HORSE	–QDVATKN**Y**SEVMEVD–
COW	–RNVATKN**Y**TEVIEVD–
CAT	–RNVATKN**Y**TEMIEVD–
DOG	–RNVATKN**Y**SEVIEVD–
HUMAN	–RNVTTKN**Y**SEVIEVD–
CHIMP	–RNVTTKN**Y**SEVIEVD–

D BRCA1

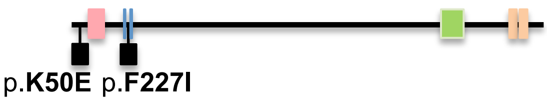

RING domain
NLS
Coiled-coil domain
BRCT domain

	p.**K50E**	p.**F227I**
XENOPUS	–FCKFCMLQLLSKKKKE–	–SDQAECE**F**SETAERT–
RAT	–FCKFCML**K**LLNQKK-G–	–AKKAACD**F**SE-GIRN–
MOUSE	–FCKFCML**K**LLNQKK-G–	–AEEAACE**F**SE-GIRN–
HORSE	–FCKFCML**K**LLNQKK-G–	–TKKAACE**F**SEKAITN–
COW		–FFPAACE**F**SEKDITN–
CAT	–FCKFCML**K**LLNQRK-G–	–AKKASCE**F**SE-DITN–
DOG	–FCKFCML**K**LLNQRK-G–	–AKKAACE**F**SG-DITN–
HUMAN	–FCKFCML**K**LLNQKK-G–	–AKKAACE**F**SETDVTN–
CHIMP	–FCKFCML**K**LLNQKK-G–	–AKKAACE**F**SETDVTN–

B RAD50

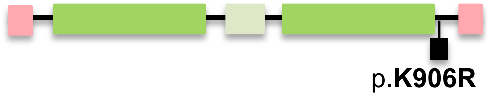

Walker Motif
CXXC motif (hook)
Coiled-coil domain

p.**K906R**	
XENOPUS	–QCLSREI**K**EAKEQVF–
RAT	–QSLNREI**K**DAKEQIN–
MOUSE	–QSLNREI**K**DAKEQIS–
HORSE	–QSLYREI**K**DAKEQVS–
COW	–QSLHREI**K**DAKEQLS–
CAT	–QSLFREI**K**DAKEQLS–
DOG	–QSLSREI**K**DAKEQLS–
HUMAN	–QSLYREI**K**DAKEQVS–
CHIMP	--SLYREI**K**DAKEQVS–

E BRCA2

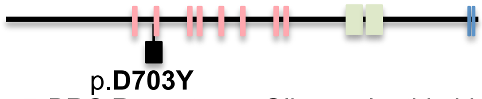

BRC Repeats
Oligonucleotide binding domain
NLS

p.**D703Y**	
XENOPUS	–MLNS-----NVSNDM–
RAT	–PERSCEN**D**QKSPKVS–
MOUSE	–PERTCEN**D**QKSPKVS–
HORSE	–QEKHCED**D**SKGQRVS–
COW	–QDKHCEDNPKSQGVS–
CAT	–QEKHCED**D**TKSQRVA–
DOG	–QEKHWED**D**AKKPRVS–
HUMAN	–QEGQCEN**D**PKSKKVS–
CHIMP	–QEGQCEN**D**PKSKKVS–

C FAN1

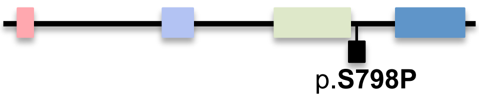

UBZ domain
SAP motif
TPR motif
VRR-Nuclease domain

p.**S798P**	
XENOPUS	–PQTGMGK**S**VFIMEEL–
RAT	–PQHGMGK**S**VFVIEG–
MOUSE	–PQHGMGK**S**VFVMES–
HORSE	–PQSGMGK**S**VFVMEA–
COW	–PQRGSGK**S**VFVLEA–
CAT	–PQRGMGK**S**VFVMEA–
DOG	–PQHGMGK**S**VFVMEA–
HUMAN	–PQRGMCK**S**VFVMEA–
CHIMP	–PQRGMCK**S**VFVMEA–

F SPIN1

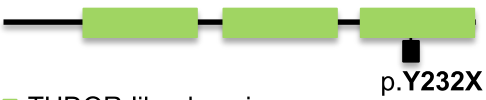

TUDOR-like domains

p.**Y232X**	
XENOPUS	–FDDDFHI**Y**VYDLVKT–
RAT	–FDDDFHI**Y**VYDLVKT–
MOUSE	–FDDDFHI**Y**VYDLVKT–
HORSE	–FDDDFHI**Y**VYDLVKT–
COW	–FDDDFHI**Y**VYDLVKT–
CAT	–FDDDFHI**Y**VYDLVKT–
DOG	–FDDDFHI**Y**VYDLVKT–
HUMAN	–FDDDFHI**Y**VYDLVKT–
CHIMP	–FDDDFHI**Y**VYDLVKT–

Supplemental Fig_S2 Legend: All amino acids as well as their surrounding sequence are highly conserved. Protein structures of (A) MRE11, (B) RAD50, (C) FAN1, (D) BRCA1, (E) BRCA2, and (F) SPIN1 with the location of the chemically-induced mutations are shown.

Supplemental_Fig_S3.pdf: Sanger sequence traces

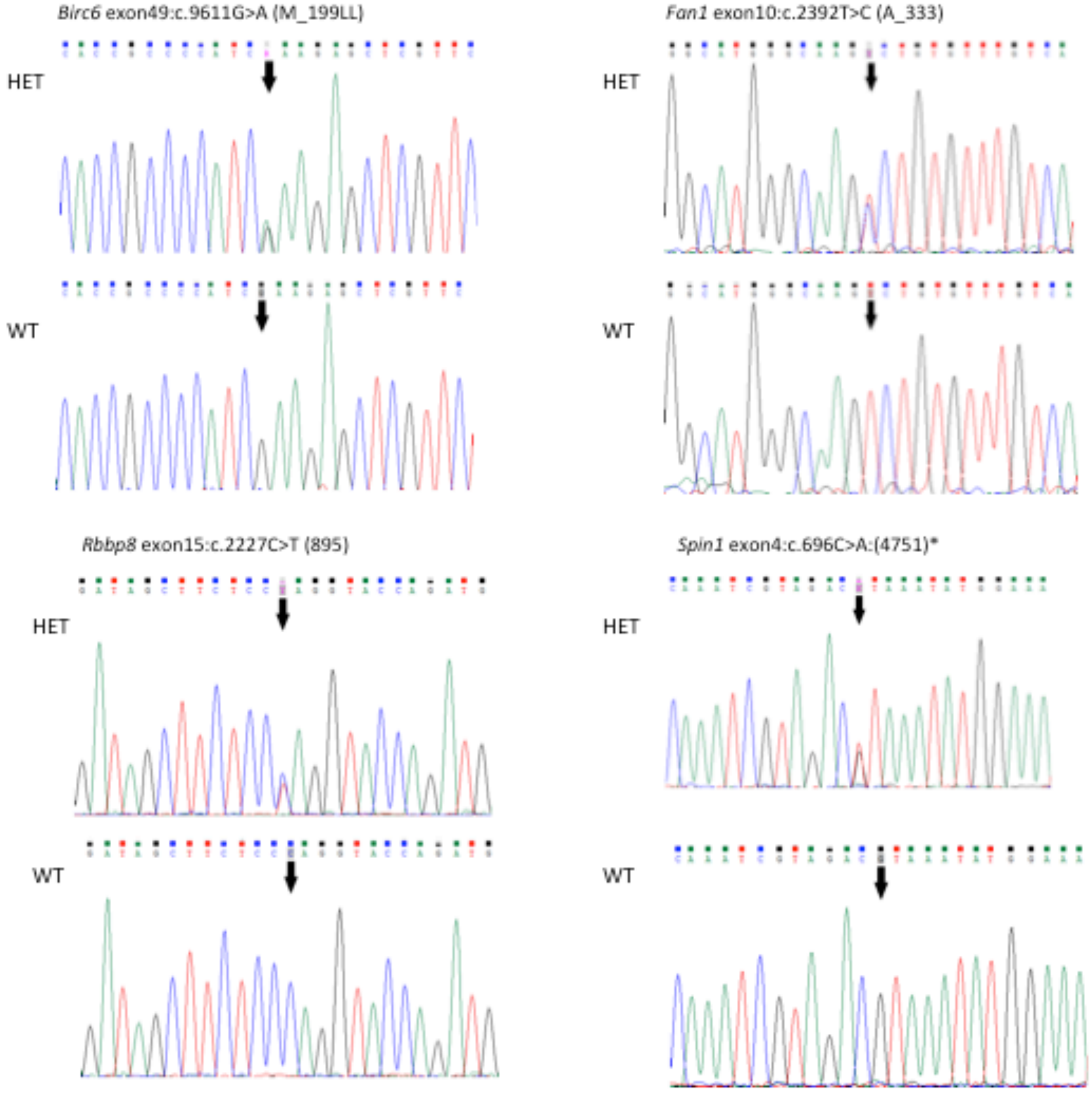

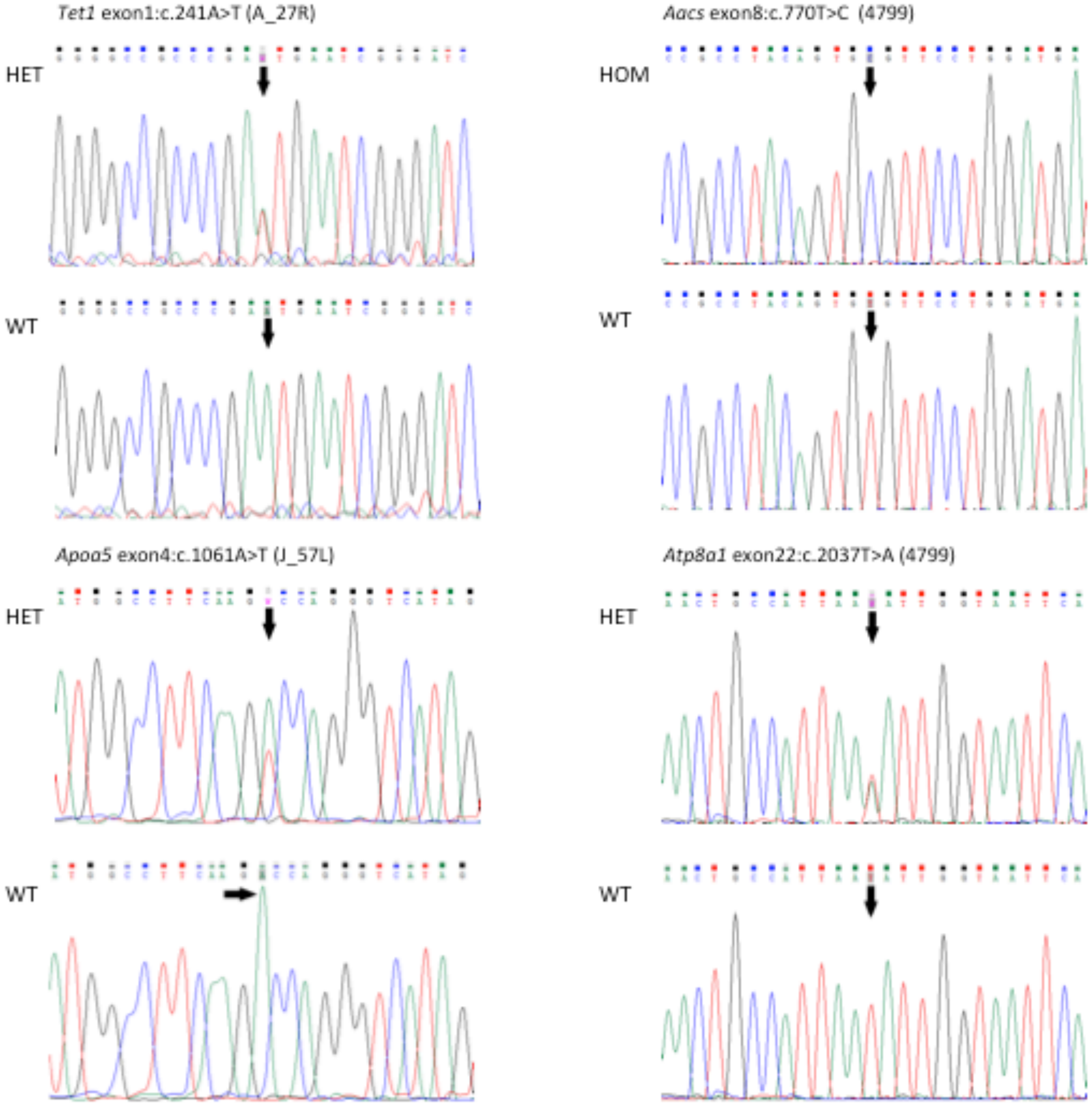

Tet1 exon1:c.241A>T (A_27R)
HET
WT
Aacs exon8:c.770T>C (4799)
HOM
WT
Apoa5 exon4:c.1061A>T (J_57L)
HET
WT
Atp8a1 exon22:c.2037T>A (4799)
HET
WT

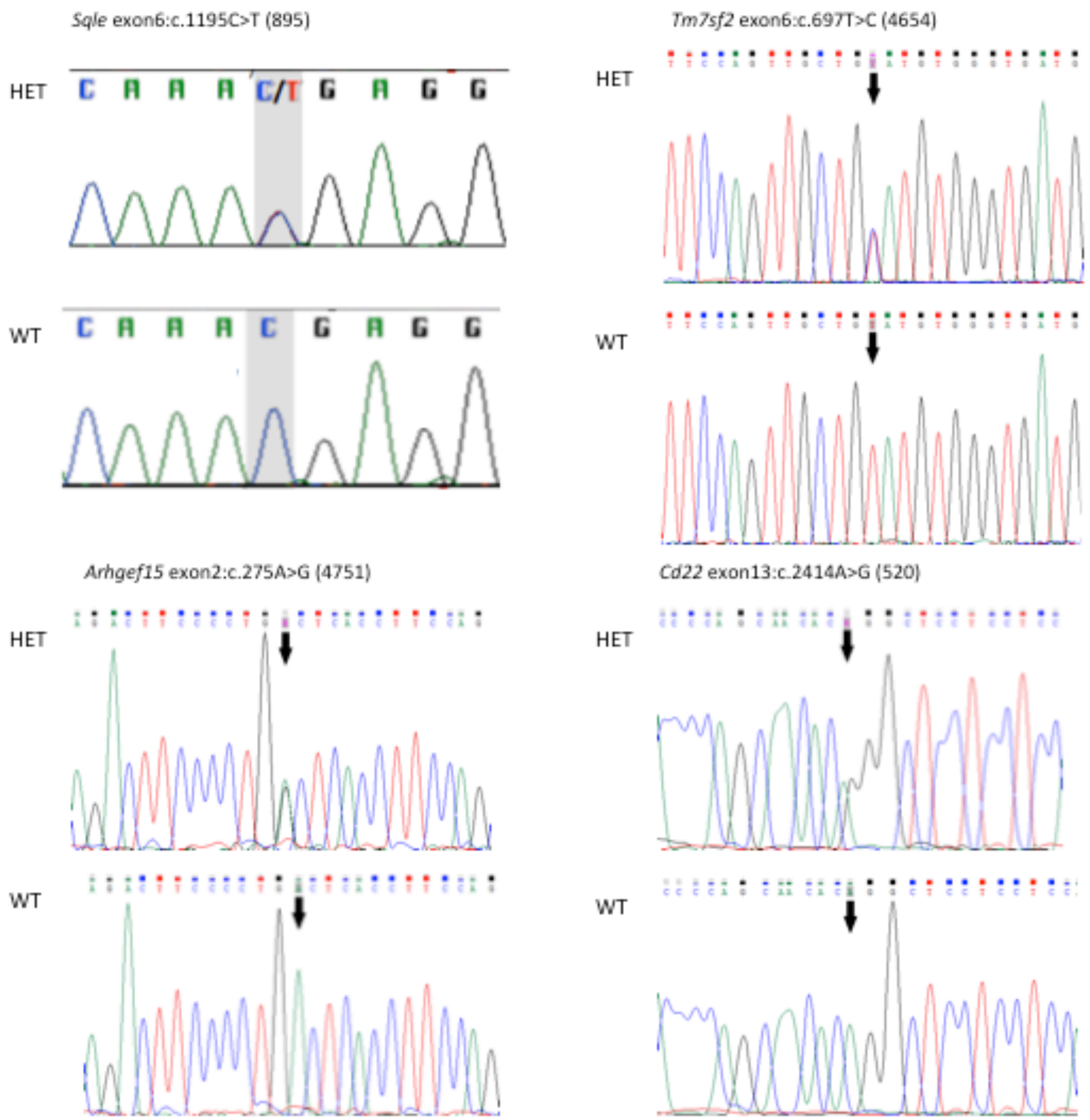

Sqle exon6:c.1195C>T (895)
HET
C A A A C/T G A G G
WT
C A A A C G A G G
Tm7sf2 exon6:c.697T>C (4654)
HET
WT
Arhgef15 exon2:c.275A>G (4751)
HET
WT
Cd22 exon13:c.2414A>G (520)
HET
WT

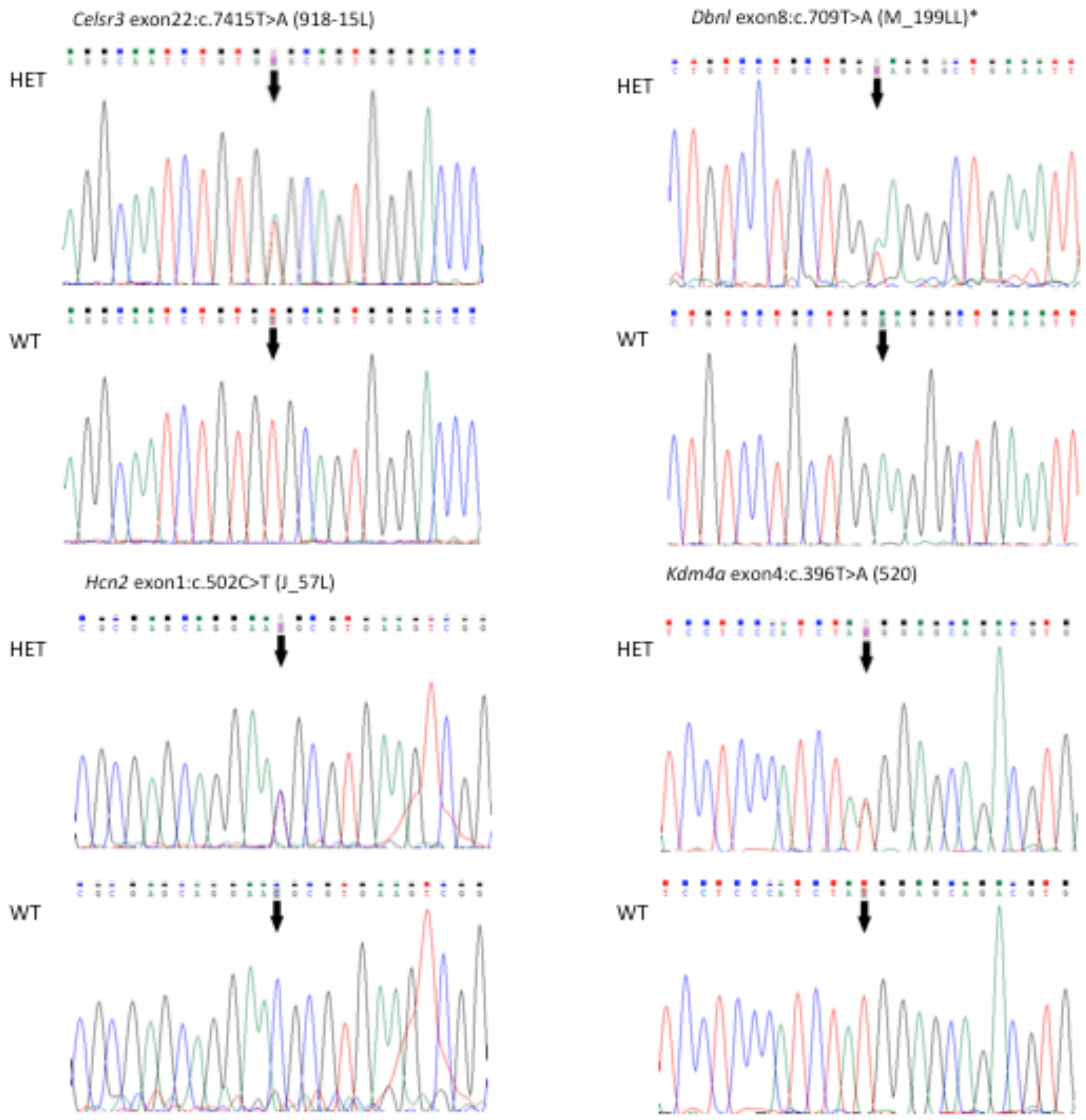

Celsr3 exon22:c.7415T>A (918-15L)
HET
WT
Dbnl exon8:c.709T>A (M_199LL)*
HET
WT
Hcn2 exon1:c.502C>T (J_57L)
HET
WT
Kdm4a exon4:c.396T>A (520)
HET
WT

Gtf3c5 exon9:c.1169A>G (4654)

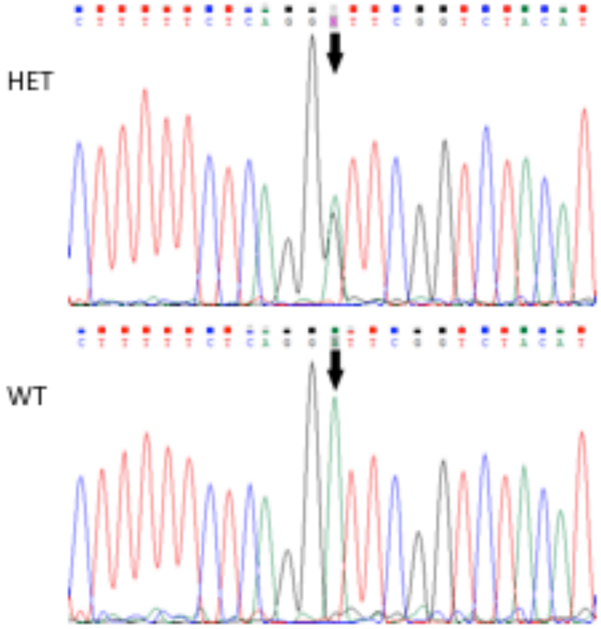

Prdm15 splicing, c.2099257392 A>G (352)*

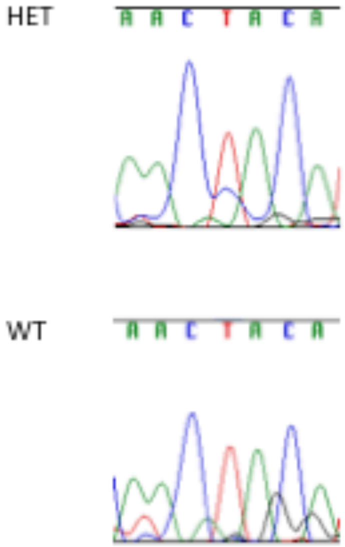

Zbtb41 exon3:c.1236C>A (M_333R)

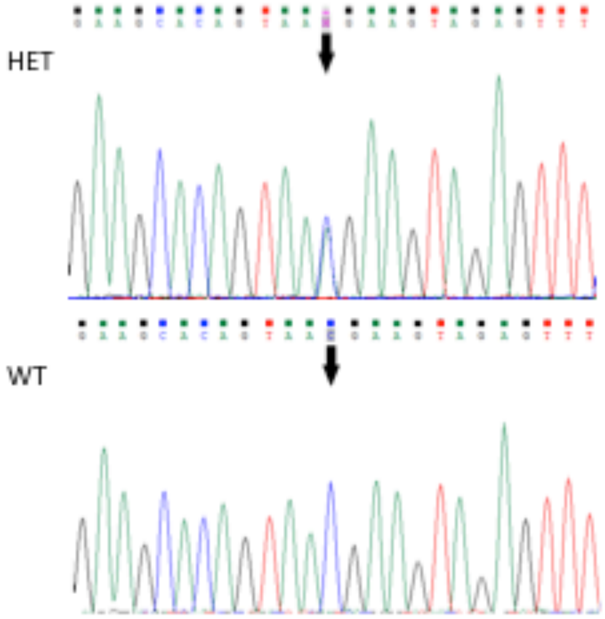

Zzz3 exon4:c.1682C>T (856)

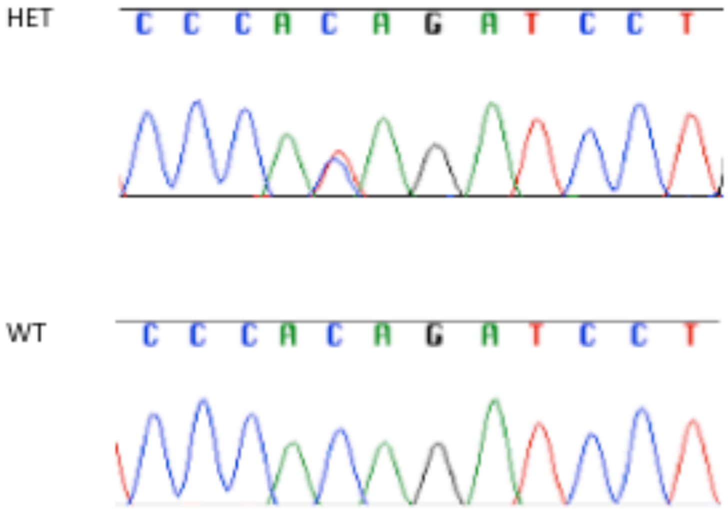

Clint1 exon12:c.1730T>C (520)

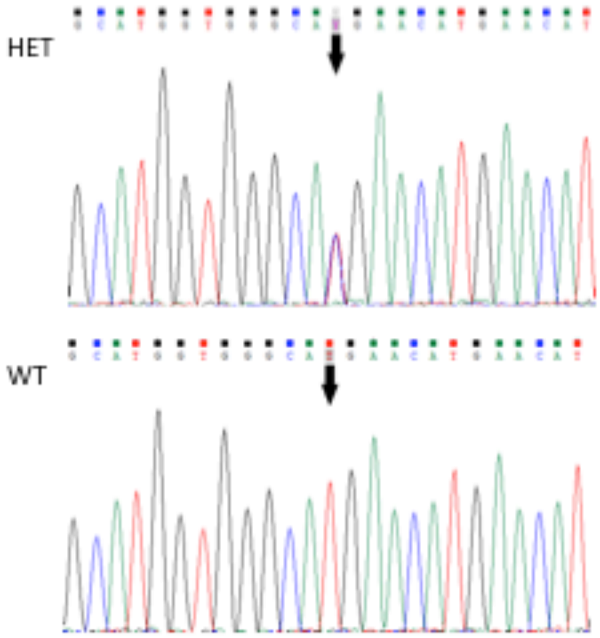

Dennd4a exon15:c.1778C>T (4654)

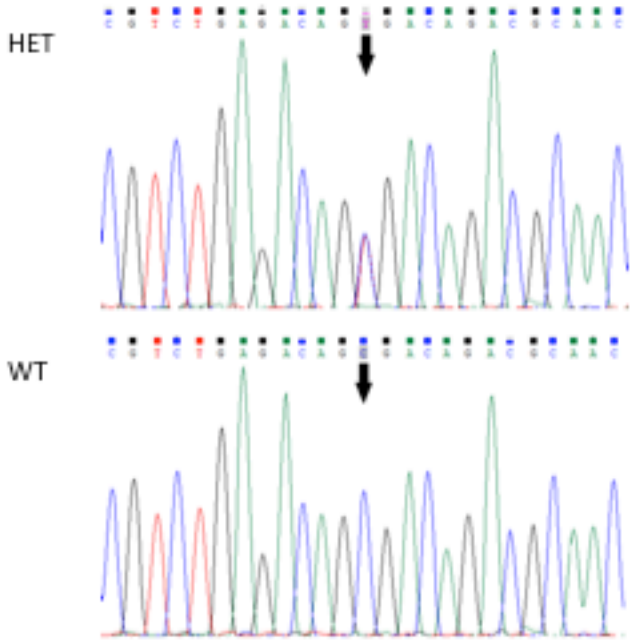

*Reverse complement used for sequencing
